# Association Between Fat‐Free Mass and Mortality: A Systematic Review and Meta‐Analysis

**DOI:** 10.1002/jcsm.70331

**Published:** 2026-07-10

**Authors:** Valerie L. Haas, Patricia Fromherz, Carmen Jochem, Hansjörg Baurecht, Volker Alt, Michael F. Leitzmann, Anja M. Sedlmeier

**Affiliations:** ^1^ Department of Epidemiology and Preventive Medicine University of Regensburg Regensburg Germany; ^2^ Department of Neurology Medbo District hospital and University hospital of Regensburg Regensburg Germany; ^3^ Department of Planetary & Public Health University of Bayreuth Bayreuth Germany; ^4^ Department for Trauma Surgery University Hospital Regensburg Regensburg Germany; ^5^ Center for Translational Oncology University Hospital Regensburg Regensburg Germany; ^6^ Bavarian Cancer Research Center (BZKF) Regensburg Germany

**Keywords:** body composition, body fat distribution, leanness, meta‐analysis, mortality

## Abstract

**Background:**

Body composition is a major determinant of health, yet the role of fat‐free mass, a key component of body composition, in mortality remains unclear.

**Methods:**

A PRISMA‐guided systematic review and meta‐analysis (PROSPERO: 321722) of observational cohort studies in community‐dwelling adults was conducted. PubMed was searched from inception to 25 October 2025, and Web of Science and Embase were searched from inception to 31 July 2025. Eligible studies assessed fat‐free mass using computed tomography, dual‐energy X‐ray absorptiometry (DXA), bioelectrical impedance analysis (BIA) or anthropometry. Studies involving hospitalized participants were excluded. Maximally adjusted effect estimates were pooled using random‐effects models to calculate summary risk ratios (RRs) and 95% confidence intervals (CIs). Publication bias was assessed using graphical and statistical methods; influence analyses evaluated robustness; E‐values quantified potential unmeasured confounding; and meta‐regression explored between‐study heterogeneity.

**Results:**

Of 7741 screened records, 49 studies met the inclusion criteria (1 149 807 participants; 83 798 deaths). Low versus high fat‐free mass was associated with higher all‐cause mortality (RR: 1.42, 95% CI: 1.30–1.55). Trim‐and‐fill analysis indicated publication bias (adjusted RR: 1.31, 95% CI: 1.20–1.44). Sensitivity analyses confirmed robustness (leave‐one‐out RR range: 1.39–1.43; E‐value: 2.19). Associations were consistent across age (*p*_difference; within‐study_ = 0.133), geographic region (*p*_difference_ = 0.983) and cause of death (*p*_difference for cardiovascular diseases; within‐study_ = 0.240, *p*_difference for cancer; within‐study_ = 0.136). Effect sizes varied by sex (men RR: 1.56, 95% CI: 1.32–1.85; women RR: 1.25, 95% CI: 1.11–1.39; *p*_difference_ < 0.0001) and measurement method: strongest for calf circumference (RR: 2.19, 95% CI: 1.50–3.20), moderate for DXA (RR: 1.52, 95% CI: 1.29–1.79) and weakest for BIA (RR: 1.23, 95% CI: 1.07–1.41).

**Conclusions:**

Low fat‐free mass is associated with a 42% higher risk of all‐cause mortality in community‐dwelling adults. Routinely assessing fat‐free mass provides clinical value in identifying high‐risk individuals and informing preventive care strategies.

**Abbreviations**
BIA: bioelectrical impedance analysis
BMI: body mass index
CI: confidence interval
CT: computed tomography
CVD: cardiovascular disease
DXA: dual‐energy X‐ray absorptiometry
HR: hazard ratio
OR: odds ratio
PRISMA: Preferred Reporting Items for Systematic Reviews and Meta‐Analyses
RR: risk ratio

## 1 Background

Growing epidemiological evidence underscores the importance of body composition as a determinant of health status and a contributing factor in the aetiology of chronic disease development [1]. A common approach to assessing body composition is the two‐compartment model, which divides total body mass into fat mass and fat‐free mass. Whereas muscle mass constitutes the largest proportion of fat‐free mass, this compartment also includes bone mass and body water [2].

Low fat‐free mass has been associated with increased mortality, particularly from noncommunicable diseases such as cardiovascular disease (CVD) and cancer [1, 2, 3, 4, 5]. In 2021, noncommunicable diseases accounted for nearly 75% of all nonpandemic‐related deaths worldwide [6], underscoring the need to identify modifiable risk factors and strengthen prevention strategies.

Body composition is typically inferred from body mass index (BMI), which provides a practical but nonspecific measure of adiposity. BMI shows a J‐ or U‐shaped association with mortality [7], and several studies have reported lower mortality among obese patients with established disease, a finding often referred to as the ‘obesity paradox’ [8]. However, because BMI does not distinguish between fat and lean tissue, it may obscure the distinct contributions of fat mass and fat‐free mass to health and mortality [3].

Fat mass has been studied extensively and is generally associated with higher mortality [9]. In contrast, the relationship between fat‐free mass and mortality remains less clear. Numerous studies reported an inverse association between fat‐free mass and mortality [S1–S28; see Supporting Information for full references], whereas others found no significant relation [S29–S47], and some even suggested a positive association [S48]. These inconsistencies highlight the need to systematically synthesize available evidence to clarify the role of fat‐free mass in longevity and healthy ageing.

Improved knowledge of this relationship can help refine clinical screening procedures and guide preventive health strategies. Accordingly, the current systematic review and meta‐analysis investigated the association between fat‐free mass and mortality in community‐based, nonpatient cohorts. We aimed to provide a comprehensive, up‐to‐date synthesis across anthropometric measurement methods and to explore key subgroups by age, sex, geographic region and cause of death.

## 2 Methods

We conducted this systematic review and meta‐analysis in accordance with the Preferred Reporting Items for Systematic Reviews and Meta‐Analyses (PRISMA) guidelines (Table S1) [10] and registered it on PROSPERO (ID: 321722).

### 2.1 Eligibility Criteria

Studies were eligible if they met the following criteria: observational design (prospective or retrospective cohort studies), community‐based or nonpatient populations and assessment of fat‐free mass, lean body mass, lean muscle mass or appendicular lean mass. The primary outcome was all‐cause mortality. Studies were included regardless of the measurement method, provided they assessed body composition using bioelectrical impedance analysis (BIA), dual‐energy X‐ray absorptiometry (DXA), computed tomography (CT), anthropometric measures (e.g., mid‐upper arm, mid‐arm muscle, calf or thigh circumference) or validated prediction equations.

We excluded studies conducted in hospitalized populations or residents of care facilities. Additionally, studies analysing longitudinal changes in fat‐free mass were excluded, as these data were not comparable across methods and cohorts.

### 2.2 Search Strategy

We identified eligible articles through systematic searches in PubMed (inception to 25 October 2025), Web of Science and EMBASE (both inception to 31 July 2025). Search terms combined keywords related to fat‐free mass or lean mass, study design, mortality and community‐based populations (Table S2). The search was limited to articles published in English or German. Reference lists of relevant reviews and eligible studies were screened manually to identify additional records.

Three reviewers (VLH, PF and AMS) independently conducted the literature search, screened titles and abstracts and reviewed full‐text articles. Discrepancies were resolved through discussion with a fourth reviewer (CJ).

### 2.3 Data Extraction

VLH, PF and AMS extracted data including first author, publication year, journal, study design and name, geographic region, follow‐up duration, sex, total number of participants, age, number of deaths, case ascertainment method, maximally adjusted risk estimates (hazard ratios [HRs] or odds ratios [ORs] with corresponding 95% confidence intervals [CIs]), confounding variables and body composition measurement technique.

Fat‐free mass was expressed using various metrics, including total fat‐free mass (kg), fat‐free mass index (kg/m^2^), appendicular skeletal muscle mass (kg), appendicular skeletal muscle mass index (kg/m^2^), skeletal muscle mass index (kg/m^2^), lean mass (kg or %), lean mass index (kg/m^2^), muscle area (cm^2^) and limb circumferences (arm, calf or thigh; cm).

### 2.4 Statistical Methods

For each study, a single risk estimate was extracted, prioritizing the maximally adjusted model. When multiple measurement methods were available, the most valid estimate was selected according to a predefined hierarchy: CT > DXA > BIA > anthropometric circumferences > prediction equations. CT and DXA were considered as reference standards, whereas BIA is sensitive to hydration status, and anthropometric measures can overestimate fat‐free mass. When multiple body regions were reported, total lean mass was preferred over appendicular measures, and leg DXA was prioritized over arm DXA. Sex‐combined estimates were used when available; otherwise, sex‐specific estimates were pooled.

For categorical exposures, the lowest category was compared with the highest. If the reference group corresponded to the 50th percentile, the lowest percentile was used as the exposure group. To ensure comparability, all estimates were standardized so that high fat‐free mass represented the reference and low fat‐free mass the exposure. When necessary, reciprocal estimates were calculated with reversed CIs.

The main meta‐analysis included studies reporting HRs; studies reporting ORs were analysed separately. Outcomes were expressed as the natural logarithm of observed risk ratios (log(rr_i_)), with sampling variances calculated as the squared standard error. The standard error was derived as log (log(CI.upper) − log(CI.lower))/(2 × 1.96) [11]. Random‐effects models were applied using restricted maximum likelihood (REML) estimation, and the Knapp and Hartung adjustment was applied to account for uncertainty in the estimation of the overall effect [11]. Between‐study heterogeneity was quantified using *τ* ^2^ and the *I* ^2^ statistic [12].

Pre‐specified meta‐regression and subgroup analyses examined potential effect modifiers, including sex (men, women or combined), measurement method (BIA, DXA, circumferences or prediction equations), geographic region (Europe, North America, South America and Asia‐Pacific), age group (< 65 or ≥ 65 years) and cause‐specific mortality (CVD or cancer). Most studies classified CVD and cancer deaths according to the International Classification of Diseases, 10th revision (ICD‐10) [13] (see Table S3). In additional sensitivity analyses, we excluded deaths occurring within the first 2 years of follow‐up to minimize potential reverse causation. Subanalyses were performed only when data from at least three studies were available.

Risk of bias was assessed using the Cochrane Risk of Bias in Nonrandomized Studies of Exposures (ROBINS‐E) tool [14, 15]. Regarding the confounding domain, studies adjusting for age, sex, smoking, physical activity, major comorbidities and BMI or fat mass were judged to be at low risk of bias. Assessments were performed independently by two researchers (AMS and PF), with any discrepancies resolved through consensus. The robvis tool was used to visualize the risk of bias assessments [16]. Certainty of evidence was evaluated using the GRADE approach for observational studies [17].

Publication bias was assessed using funnel plots, trim‐and‐fill analysis [18], Egger's regression test [18] and Begg's rank correlation test [19]. Influence diagnostics and leave‐one‐out analyses [20] were performed to identify influential studies. The E‐value was calculated to quantify the strength of unmeasured confounding required to explain the observed association [21].

All statistical analyses were conducted using R (Version 4.1.2), with the metafor [11], robumeta [22], EValue [21] and MetaUtility [23] packages. A *p*‐value of < 0.05 was considered statistically significant.

## 3 Results

### 3.1 Study Selection

**FIGURE 1:**
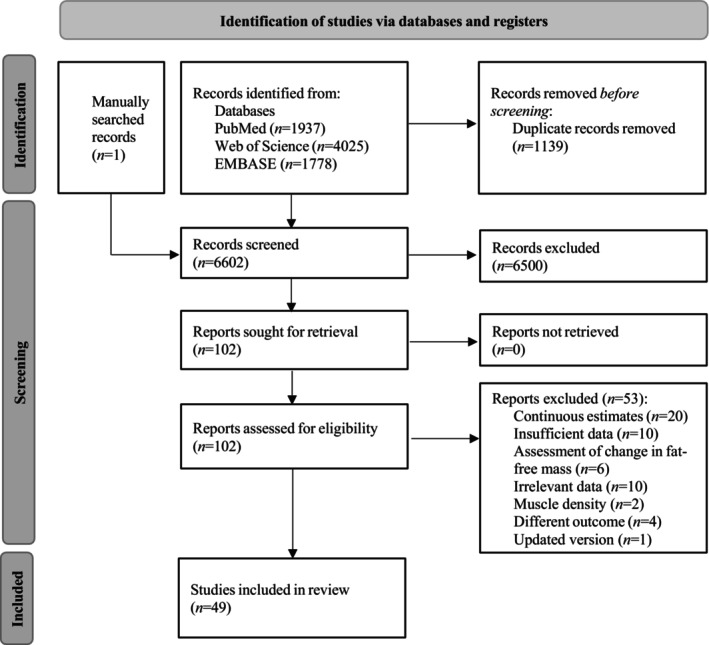
PRISMA flow chart of systematic literature search.

A total of 7741 records were identified: 1937 through PubMed, 4025 through Web of Science, 1778 through Embase and one through a manual reference list search. After removing 1139 duplicates, 6602 records remained for title and abstract screening. Of these, 6500 were excluded, leaving 102 full‐text articles for eligibility assessment. Fifty‐three studies were excluded after full‐text review (Figure 1). Wu et al. [S44, S49] published two analyses based on the same cohort; one was retained for the main analysis, and the other contributed to the sex‐specific subanalysis. In total, 49 studies met the inclusion criteria and were included in the systematic review and meta‐analysis. All but one study reported HRs; one reported ORs [S39].

### 3.2 Study Characteristics

This meta‐analysis included 1 149 807 participants and at least 83 798 deaths. Most studies used maximally adjusted estimates that included between one and 20 covariates. The ten most frequently adjusted variables were age, sex, smoking status, alcohol consumption, physical activity, BMI, education or socio‐economic status, marital status, comorbidities (e.g., CVD, diabetes and cancer) and ethnicity (Tables 1 and S3).

**TABLE 1:** Characteristics of included studies.

Author, year, country [reference]	Participants/deaths	Age in years	Fat‐free mass measurement	Adjustment variables
Kim D, 2024, USA [S19]	16 839/2109	> 20	Appendicular skeletal muscle index in kg/m^2^ by DXA	Age, sex, race, smoking status, alcohol consumption, estimated glomerular filtration rate, central obesity, history of cancer
Liu CA, 2023, USA [S1]	5052/826	20–59	Total lean mass in g by DXA	Age, sex, race/ethnicity, education level, marital status, family income–poverty ratio level, hypertension, coronary heart disease, diabetes, cancer, smoke, covered by health insurance, alcohol, BMI, waist, muscle strengthening activities, Healthy Diet Index score
Bernabe‐Ortiz A, 2023, Peru [S46]	3216/172	≥ 30	Skeletal muscle mass in kg by BIA	Age, sex, education level, socio‐economic level, daily smoking, alcohol use, physical activity levels and body mass index
Chang CS, 2023, Taiwan [S25]	224/NA	≥ 65	Skeletal muscle mass index in kg/m^2^ by BIA	Exercise, gender, age, socio‐economic status, smoking, alcohol consumption and Charlson comorbidity index
Liu J, 2023, USA [S23]	17 735/3446	≥ 65	Circumference measurements in cm	Age, sex, race/ethnicity, education
Ying Z, 2023, Taiwan [S26]	422 230/11 892	≥ 20	Fat‐free mass index in kg/m^2^ by BIA	Fat mass index, age, gender, marital status, education, occupation, smoking status, drinking status, physical activity and dietary intake (light vegetable, dark vegetable, fruit and meat intake)
Camargo Pereira C, 2022, Brazil [S20]	418/147	≥ 60	Arm muscle circumference in cm^2^	Age, sex, skin colour, education, socio‐economic class, marital status, smoking, alcohol consumption, physical activity, consumption of fruits and vegetables
Landi F, 2022, Italy [S21]	346/245	≥ 80	Appendicular skeletal muscle in kg	Age, gender, activities of daily living impairment, cognitive impairment, body mass index, C‐reactive protein and interleukin‐6
Li C, 2022, Taiwan [S22]	641/198	≥ 65	Skeletal muscle index in kg/m^2^ by DXA	Age, sex, education, marital status, smoking, alcohol drinking, physical activity, exercising programme, hypertension, diabetes mellitus, heart disease, stroke, cancer, cognitive impairment, fasting
Tabara Y, 2022, Japan [S47]	3582/189	≥ 65	Skeletal muscle index in kg/m^2^ by BIA	Age, BMI, history of cardiovascular disease, history of cancer, current smoking, systolic blood pressure, albumin, haemoglobin A1c, high‐density lipoprotein cholesterol, low‐density lipoprotein cholesterol, C‐reactive protein
Wu M, 2022, China [S24]	23 290/739	38–88	Appendicular muscle mass index in kg/m^2^ by BIA	Sex, educational attainment, marital status, occupation, household income, smoking status, alcohol consumption, levels of physical activities, scores of dietary patterns, prevalent hypertension, prevalent diabetes, prevalent COPD
Liu M, 2022, USA [S11]	55 818/10 408	≥ 18	Predicted lean mass in kg by anthropometric prediction equations	Age, sex, height, race/ethnicity, education level, marital status, smoking status, history of hypertension and diabetes, leisure physical activity level, HDL cholesterol, total cholesterol, predicted fat mass
Knowles R, 2021, UK [S48]	356 590/15 844	40–69	Appendicular skeletal muscle mass in kg by BIA	Age, height, Townsend deprivation index, education, smoking, alcohol intake, physical activity, oily fish intake, fruit and vegetable intake, saturated fat intake, diabetes, cancer history, menopause (women)
Sedlmeier AM, 2021, Germany and USA [S14]	16 155/1347	25–74, 20–49, 20–79	Fat‐free mass index in kg/m^2^ by BIA	Age, sex, cohort, ethnicity, baseline history of diabetes, education, smoking, physical activity, alcohol intake, fat mass index
Cawthon P, 2021, USA [S32]	1400/197	77–101	Appendicular lean mass in kg by DXA	Age, ethnicity, clinical centre, alcohol, smoking, comorbidities, physical activity, per cent fat, exhaustion, cognitive function, self‐reported health status, weight change, weight, height, strength and physical performance (chair stand, gait speed and grip strength)
He L, 2021, China [S8]	17 717/167	≥ 18	Mid‐upper arm circumference in cm	Age, sex, BMI, marital status, education, income, region, urbanization index, smoking, alcohol, physical activity, total energy intake, fat intake, protein intake, carbohydrate intake, blood pressure (systolic, diastolic), diabetes, TSF thickness, subcutaneous fat
Fernandes DPD, 2021, Brazil [S6]	796/197	≥ 60	Calf circumference in cm	Age, sex, education, quality of diet, physical activity, smoking, BMI
Soerensen TIA, 2020, Denmark [S16]	1951/486	35–65	Fat‐free mass index in kg/m^2^ by BIA	Age, sex, smoking, alcohol intake, physical activity, education
Seino S, 2020, Japan [S15]	966/128 (men) 1011/75 (women)	65–74; ≥ 75	Fat‐free mass index in kg/m^2^ by BIA	Age, FM, study area, year, alcohol, smoking, hypertension, CVD (stroke, heart disease), diabetes, cancer, cholesterol level, hypoalbuminaemia, anaemia, chronic kidney disease
Costanzo L, 2020, Italy [S35]	535/56	≥ 65	Skeletal muscle index in kg/m^2^ by BIA	Age, sex, BMI, marital status, education and comorbidities
Oh H, 2020, Korea [S41]	17 284/1072	20–95	Skeletal muscle index in kg/m^2^ by DXA	Marital status, residence, income, occupation, smoking, alcohol, predicted fat mass, height
Larsen B, 2020, USA [S37]	946/118 (men) 955/119 (women)	45–85	Abdominal muscle area in cm^2^/m^2^ by CT	Age, ethnicity, height, diabetes, systolic blood pressure, CVD medication (antihypertensive medication, statin use), total cholesterol, HDL cholesterol, smoking, cancer, kidney function, physical activity, sedentary time, visceral fat, BMI
De Almeida Roediger M, 2019, Brazil [S2]	1504/769	≥ 60	Mid‐upper arm circumference in cm; calf circumference in cm	Age, sex, marital status, education, working status, income, alcohol, physical activity, smoking, hypertension, CVD, lung disease, stroke, cancer, number of diseases, MMSE, geriatric depression scale
Wang H, 2019, China [S42]	238/132 (men) 500/255 (women)	90–105	Skeletal muscle index in kg/m^2^ by anthropometric equations	Age, smoking, alcohol, cognitive impairment (MMSE), disability
Loprinzi PD, 2018, USA [S40]	1079/277	50–85	Leg lean mass in g by DXA	Age, sex, race–ethnicity, relative protein intake, carbohydrate intake, fat intake, mean arterial pressure, smoking status
Li R, 2018, USA [S39]	4449/NA	≥ 50	Appendicular lean mass in kg by DXA	Age, sex, ethnicity, BMI, smoking, alcohol, education, physical activity (LTPA), sedentary time, cardiovascular disease, diabetes, cancer, chronic obstructive lung disease, chronic kidney disease
Lee DH, 2018, USA [S38]	38 006/12 356	40–75	Predicted lean mass in kg by anthropometric prediction equations	Age, ethnicity, family history of CVD, family history of cancer, physical activity, alcohol consumption, total energy intake, smoking, Alternate Healthy Eating index
Batsis JA, 2017, USA [S3]	4984/1901	≥ 60	Appendicular lean mass in kg by DXA	Age, sex, ethnicity, poverty income ratio, smoking, diabetes, CVD (congestive heart failure, coronary heart disease), nonmelanoma skin cancer, arthritis, physical activity
Pasco JA, 2017, Australia [S13]	750/190	50–92	Appendicular lean mass as *t*‐scores by DXA	Age, weight, height, BMI, mobility, polypharmacy
Balogun S, 2017, Australia [S29]	1041/145	51–81	Appendicular lean mass index in kg/m^2^ by DXA	Age
Wu L, 2017, USA [S44]	11 958/NA	20–90	Mid‐arm circumference in cm	Age, sex, ethnicity, BMI, serum triglycerides, serum aspartate transaminase, serum HDL, serum glucose, CRP, serum uric acid, serum total bilirubin, systolic blood pressure, smoking, diabetes, physical activity
Wu L, 2017, USA [S49]	3373/1396 (men) 3396/1097 (women)	40–90	Mid‐arm muscle circumference in cm	Age, ethnicity, BMI, waist circumference, serum total cholesterol, serum HDL, serum glucose, CRP, serum uric acid, serum total bilirubin, systolic blood pressure, smoking, diabetes, congestive heart failure, serum albumin, marital status, number of prescription medications taken
Cheung CL, 2016, USA [S34]	2304/330	≥ 65	Appendicular lean mass index in kg/m^2^ by DXA	Age, sex, ethnicity, smoking, alcohol, CRP, triglycerides, HDL cholesterol, eGFR
Zong G, 2016, USA [S45]	9471/682	≥ 20	Fat‐free mass index in kg/m^2^ by DXA	Age, sex, ethnicity, education, marital status, family income‐to‐poverty ratio, family history of chronic diseases (diabetes, hypertension, stroke or angina), physical activity, smoking status, alcohol consumption, BMI
Bea JW, 2015, USA [S31]	10 525/1762	50–79	Lean mass in % by DXA	Age, age at menopause, physical activity, diet quality, ethnicity, smoking, alcohol, hormone use
Srikanthan P, 2014, USA [S17]	3659/2012	≥ 55	Skeletal muscle mass index in kg/m^2^ by BIA	Age, sex, ethnicity, central obesity, smoking, cancer, CRP, hypertension, cholesterol (HDL and total), HOMA‐IR, HbA1c, diabetes, prediabetes, serum creatinine
Chuang SY, 2014, Taiwan [S5]	1512/506	≥ 65	Skeletal muscle mass index in kg/m^2^ by BIA	Age, sex, BMI, smoking, alcohol, physical activity, CRP, eGFR, number of comorbidities
Batsis JA, 2014, USA [S30]	1569/792	≥ 60	Lean mass in kg by BIA	Age, sex, ethnicity, smoking, CVD, self‐reported health, hypertension, diabetes, dyslipidaemia, physical activity
Chen Y, 2014, Bangladesh [S33]	19 575/744	≥ 18	Mid‐upper arm circumference in cm	Age, sex, BMI, education level, betel use, smoking status, baseline systolic blood pressure
Genton L, 2013, Switzerland [S7]	203/58	≥ 65	Fat‐free mass index in kg/m^2^ by BIA	Age, sex, FMI, physical activity, Charlson index
Bites AC, 2013, Chile [S4]	75/23	61–91	Lean mass/height in g/cm by DXA	Age, hand grip strength, 12‐min walking capacity, total lean mass/height^2^
Tsai AC, 2011, Taiwan [S18]	4191/566	≥ 53	Mid‐arm circumference in cm; calf circumference in cm	Age, sex, smoking, physical activity
Landi F, 2010, Italy [S10]	357/146	≥ 80	Mid‐arm muscle circumference in cm	Age, sex, living alone, sensory impairments, albumin, cholesterol, body mass index
Heitmann BL, 2009, Denmark [S9]	1436/257	35–65	Thigh circumference in cm	Age, smoking, physical activity, education, body fat percentage, body height, BMI, waist circumference, alcohol, systolic blood pressure, total cholesterol, triglycerides
Wannamethee SG, 2007, UK [S43]	4107/713	60–79	Fat‐free mass index in kg/m^2^ by BIA	Age, social class, physical activity, alcohol intake, smoking
Dolan CM, 2007, USA [S36]	8029/945	≥ 65	Lean mass in kg by BIA	Age, self‐reported health, grip strength, nonthiazide diuretic use, femoral neck bone mineral density
Miller MD, 2002, Australia [S12]	1396/579	≥ 70	Corrected arm muscle area in cm^2^	Age, sex, marital status, smoking, self‐rated health, activities of daily living, cancer, CVD, diabetes, hypertension, respiratory disease, depression (CESD), cognitive impairment (MMSE)
Moon S, 2025, Korea and USA [S27]	8036/1058 and 14 449/1045	48.0 ± 17.1 and 48.5 ± 16.4	Appendicular skeletal mass index in kg/m^2^ by DXA	Age, sex, ethnicity, smoking status, alcohol consumption, history of cancer, estimated glomerular filtration rate, dyslipidaemia and HTN at baseline
Cheng Y, 2025, USA [S28]	21 938/2885	20–85	Appendicular skeletal muscle mass index in kg/m^2^ by DXA	Age, sex, ethnicity, marital status, educational level, family income‐to‐poverty ratio, BMI, history of chronic diseases

Abbreviations: BIA: bioelectrical impedance analysis; DXA: dual‐energy X‐ray absorptiometry.

Fat‐free mass was quantified using various metrics. Ten studies applied anthropometric circumferences (arm: *n* = 5, thigh: *n* = 1, calf: *n* = 1, calf and arm: *n* = 2, unspecified: *n* = 1). Eight studies reported skeletal muscle mass index, and seven reported fat‐free mass index. Lean mass was expressed as absolute mass in kilograms in five studies, as a percentage of body mass in one study, as total lean mass in grams in one study, as leg lean mass in grams in one study and as lean mass relative to height (g/cm) in one study. Six studies each reported appendicular muscle or lean mass, either as absolute values or normalized to height^2^. Finally, three studies assessed muscle area (arm: *n* = 2, abdominal region: *n* = 1).

### 3.3 Fat‐Free Mass and All‐Cause Mortality

**FIGURE 2:**
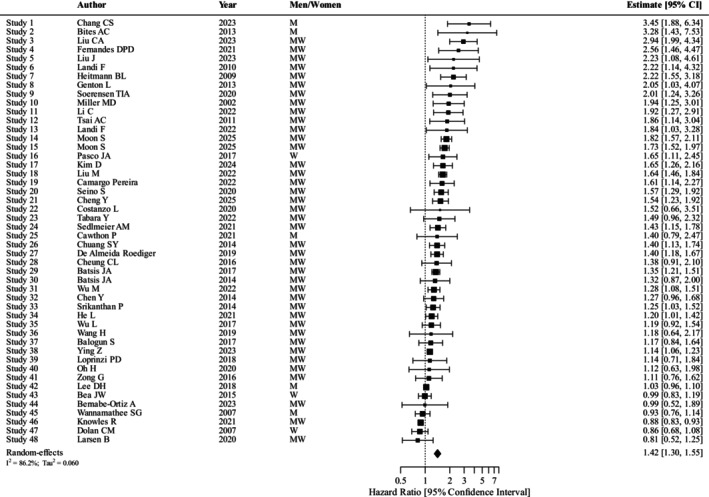
Random‐effects meta‐analysis of relative risks evaluating the association between low fat‐free mass and mortality. The black square and the respective line represent the risk estimate and the corresponding 95% confidence interval for each study. The diamond represents the summary relative risk with the corresponding 95% CI for low fat‐free mass and all‐cause mortality.

The primary random‐effects meta‐analysis (48 HR estimates) showed that low fat‐free mass was associated with higher all‐cause mortality (pooled ratio: 1.42, 95% CI: 1.30–1.55; Figure 2). Between‐study heterogeneity was substantial (*I* ^2^ = 86.22%, *p*_heterogeneity_ < 0.0001). Among individual studies, 28 reported a statistically significant positive association between low fat‐free mass and mortality [S1–S28]. One study observed the opposite pattern, with high fat‐free mass linked to increased mortality [S48]. Nineteen studies reported a non‐significant association—14 of which suggested a higher mortality with low fat‐free mass [S29, S30, S32–S35, S38–S42, S44, S45, S47], whereas five found no association or a possible positive relation between high fat‐free mass and mortality [S31, S36, S37, S43, S46]. The single study reporting ORs found a higher risk of mortality with lower lean mass (OR: 1.40, 95% CI: 0.91–2.14), although the result was not statistically significant [S39].

### 3.4 Sensitivity Analysis

Results of the risk‐of‐bias assessment are summarized in Figure S1. Among the 49 included cohort studies, seven were rated at low overall risk of bias, 39 at moderate risk and three at high risk.

Visual inspection of the funnel plot suggested asymmetry (Figure S2a). Egger's test indicated significant small‐study effects (*p* = 0.002), whereas Begg's test was not statistically significant (*p* = 0.663). The trim‐and‐fill method imputed nine potentially missing studies on the left side of the funnel plot (Figure S2b). After adjustment, the summary risk estimate was slightly attenuated from 1.42 (95% CI: 1.30–1.55) to 1.31 (95% CI: 1.20–1.44), yet the association remained statistically significant.

Influence diagnostics, including leave‐one‐out analyses, indicated minimal variation in the pooled effect estimate (pooled HRs ranged from 1.39 [95% CI: 1.28–1.52] to 1.43 [95% CI: 1.31–1.57]), suggesting that no individual study exerted disproportionate influence.

E‐value analysis indicated that an unmeasured confounder would need to be associated with both fat‐free mass and mortality by at least a factor of 2.19 to fully explain the observed association (RR: 1.42) or by 1.92 to attenuate the association to non‐significance (lower 95% CI bound: 1.30).

### 3.5 Subgroup Analysis

We conducted stratified analyses to assess the impact of sex, measurement method, geographic region and age. We also examined cause‐specific mortality and the effect of excluding early deaths.

Comparing sex‐specific estimates, the association between low fat‐free mass and higher mortality was more pronounced in men (RR: 1.56, 95% CI: 1.32–1.85) than in women (RR: 1.25, 95% CI: 1.11–1.39; Table 2). A within‐study comparison of 17 studies reporting both sexes confirmed a 16% stronger association in men than women (HR_men vs. women_: 1.16, 95% CI: 1.02–1.32, *p* = 0.021; Table 3).

**TABLE 2:** Summary risk estimates of low versus high fat‐free mass in relation to mortality, stratified by potential effect modifiers.

Subanalyses	No. of studies	Risk ratio (95% confidence interval)	*p*_difference_
Sex			
Men	22	1.56 (1.32–1.85)	< 0.001
Women	20	1.25 (1.11–1.39)	
Men and women	32	1.48 (1.34–1.63)	
Method			
Bioelectrical impedance analysis	15	1.23 (1.07–1.41)	0.043
Dual‐energy X‐ray absorptiometry	15	1.52 (1.29–1.79)	
Mid‐arm muscle circumference	9	1.43 (1.27–1.63)	
Calf circumference	6	2.19 (1.50–3.20)	
Prediction equations	8	1.34 (1.10–1.64)	
Geographic region			
Europe	10	1.53 (1.16–2.01)	0.983
North America	18	1.29 (1.12–1.50)	
South America	5	1.66 (1.02–2.72)	
Asia‐Pacific	16	1.44 (1.27–1.63)	
Age			
< 65 years	10	1.96 (1.54–2.51)	0.022
≥ 65 years	22	1.45 (1.25–1.67)	
Cause‐specific mortality			
Cardiovascular diseases	14	1.51 (1.29–1.77)	0.850
Cancer	9	1.17 (0.83–1.65)	0.120
Early deaths			
Exclusion of deaths within at least 2 years	6	1.22 (0.97–1.54)	0.360
Risk estimate			
Hazard ratio	47	1.42 (1.30–1.55)	
Odds ratio	1	1.40 (0.91–2.14)	

**TABLE 3:** Within‐study comparison: Summary risk estimates of low versus high fat‐free mass in relation to mortality, stratified by potential effect modifiers.

Within‐study comparison^a^	No. of studies	Risk ratio (95% confidence interval)	*p*
Sex			
Men vs. women	17	1.16 (1.02–1.32)	0.021
Age			
≥ 65 vs. < 65 years	4	0.78 (0.55–1.10)	0.133
Cause‐specific mortality			
CVD vs. all‐cause	12	0.94 (0.85–1.04)	0.240
Cancer vs. all‐cause	8	0.88 (0.74–1.05)	0.136
Early deaths			
Early deaths excluded vs. main analysis	6	0.97 (0.91–1.05)	0.434

^a^ Within‐study analysis: restricted to studies reporting both relevant estimates.

Abbreviation: CVD: cardiovascular disease.

Associations were observed across all measurement methods but varied in magnitude. The strongest association was found for calf circumference (RR: 2.19, 95% CI: 1.50–3.20), followed by DXA (RR: 1.52, 95% CI: 1.29–1.79) and upper‐arm circumference (RR: 1.43, 95% CI: 1.27–1.63). Associations were weaker for prediction equations (RR: 1.34, 95% CI: 1.10–1.64) and BIA (RR: 1.23, 95% CI: 1.07–1.41; see Table 2).

Across all geographic regions, low fat‐free mass was consistently linked to higher mortality, with no significant regional differences (*p* = 0.983). The association was strongest in South America (RR: 1.66, 95% CI: 1.02–2.72), followed by Europe and Asia‐Pacific, and weakest in North America (RR: 1.29, 95% CI: 1.12–1.50; see Table 2).

Across all studies (age range 18–105 years), the association between low fat‐free mass and higher mortality was stronger among participants < 65 years (RR: 1.96, 95% CI: 1.54–2.51) than among those ≥ 65 years (RR: 1.45, 95% CI: 1.25–1.67), with a significant between‐group difference (*p* = 0.022; Table 2). However, in within‐study comparisons limited to cohorts reporting both age strata (*n* = 4), the difference was not statistically significant (HR_≥ 65 years/< 65 years_: 0.78, 95% CI: 0.55–1.10; *p* = 0.133; Table 3).

Cause‐specific analyses generally mirrored the main findings. Low fat‐free mass was associated with higher cardiovascular mortality (RR: 1.51, 95% CI: 1.29–1.77). For cancer mortality, the association was weaker and less precise (RR: 1.17, 95% CI: 0.83–1.65). Formal comparisons between all‐cause mortality and cause‐specific mortality showed no significant differences (*p* = 0.121; Tables 2 and 3).

To address potential bias from undiagnosed diseases leading to early death, we repeated the analysis including only studies that excluded deaths within the first 2 years of follow‐up. The association was attenuated (RR: 1.22, 95% CI: 0.97–1.54), but a formal comparison with the main analysis showed no significant difference (HR: 0.97, 95% CI: 0.91–1.04, *p* = 0.360; within‐study *p* = 0.434; Tables 2 and 3).

### 3.6 Meta‐Regression and Exploration of Heterogeneity

To further investigate between‐study heterogeneity, we performed univariate and multivariate meta‐regression analyses. The association between fat‐free mass and mortality was not significantly modified by the year of publication (*p* = 0.208, *R* ^2^ = 6.38%; Figure S3), sex (*p* = 0.189, *R* ^2^ = 9.87%; Figure S4) or the specific measurement device used (*p* = 0.132, *R* ^2^ = 10.23%; Figure S5). A multivariate meta‐regression model including all three moderators simultaneously accounted for 18.72% of the heterogeneity, yet the overall test for moderators remained non‐significant (*F*(7, 39) = 1.80, *p* = 0.116; Table S5).

## 4 Discussion

This meta‐analysis, comprising more than 1 million participants, provides the most comprehensive synthesis to date of the association between fat‐free mass and mortality in community‐based adults. The findings demonstrate that individuals with low fat‐free mass have a 42% higher risk of all‐cause mortality than those with higher fat‐free mass. The association remained robust across age groups, sex, measurement methods and geographic regions. Collectively, these findings clarify the contribution of fat‐free mass to health and longevity and highlight its relevance for preventive strategies in clinical and public health practice.

Previous meta‐analyses examining fat‐free mass and mortality were limited by their focus on specific populations. The most recent meta‐analysis, including 11 studies, reported a 30% higher all‐cause mortality risk among middle‐aged and older adults [24]. Similarly, a pooled analysis of nine studies restricted to adults ≥ 65 years showed an inverse association between appendicular skeletal muscle index and mortality [25]. Another meta‐analysis up to 2017 found a comparable inverse association between low lean mass and increased mortality [26] but included populations with severe illnesses, such as cancer, which can influence fat‐free mass [27].

In contrast, our study restricted analyses to community‐dwelling, nonpatient populations, minimizing bias related to disease‐related wasting and enhancing relevance for primary prevention. By incorporating 28 additional studies published since 2017, our study substantially expands the available evidence base and provides the clearest estimate to date of the fat‐free mass–mortality association.

Men generally have greater fat‐free mass than women, whereas women have higher fat mass and a larger subcutaneous fat fraction [28]. Our findings reveal that the inverse association between fat‐free mass and mortality is significantly stronger in men. These results refine earlier evidence that found minimal or no sex difference [24] and imply that loss of muscle and other lean tissue may be particularly detrimental in men, possibly because fat‐free mass contributes proportionally more to metabolic homeostasis.

Differences in effect estimate magnitude by measurement technique likely reflect differences in construct validity. Circumference measures produced larger pooled estimates, but these methods tend to overestimate fat‐free mass [29]. BIA‐based estimates were weaker and more sensitive to age, degree of adiposity and ethnicity [30]. DXA, a more precise method [30], produced associations of intermediate magnitude, lending credibility to the overall findings.

Low fat‐free mass was consistently associated with higher mortality across all world regions, with no significant regional differences. Apparent variation by region likely reflected the measurement techniques predominantly used in specific areas or population‐specific anthropometric equations. Ethnic variation in body composition may also contribute [31], but these factors did not materially affect the overall direction of the association.

Although most included studies focused on older adults (≥ 65 years), younger participants (< 65 years) showed a stronger association between low fat‐free mass and all‐cause mortality in between‐study comparisons. This pattern aligns with a prior meta‐analysis reporting stronger associations in adults aged 45–65 years [24]. However, within‐study age comparisons showed no statistically significant age difference, suggesting that the apparent contrast may reflect measurement variability rather than true effect modification. Circumference‐based methods, which were more common in younger cohorts, may exaggerate risk estimates due to overestimation of fat‐free mass [29].

Notwithstanding, biological mechanisms could also underlie these age patterns. Lower fat‐free mass earlier in life has been linked to adverse cardiometabolic profiles. In youth, higher fat‐free mass is associated with lower levels of total and low‐density lipoprotein cholesterol, glucose and reduced insulin resistance. Obese youth with high fat‐free mass show cardiometabolic profiles comparable to normal‐weight peers [32]. These observations underscore the importance of preserving muscle mass throughout life and suggest that preventive measures to maintain fat‐free mass should begin early.

Low fat‐free mass was associated with higher cardiovascular mortality, consistent with literature linking low muscle mass to adverse cardiometabolic profiles [4] and increased risk of major cardiovascular events [33]. Potential pathways include lower cardiorespiratory fitness, as individuals with reduced fitness, regardless of adiposity, have an elevated risk of cardiovascular death [34].

For cancer mortality, the association was weaker and not statistically significant, though it did not differ significantly from the overall inverse association. Greater muscle mass has been linked to improved cancer survival and reduced treatment‐related toxicity [35]. Experimental data indicate that exercise‐induced interleukin‐6 can inhibit tumour initiation and progression by promoting natural killer cell redistribution in tumour tissue [36], and myokine‐mediated anti‐inflammatory effects may counteract cancer‐related low‐grade systemic inflammation [37]. Future studies should examine site‐specific cancer outcomes and incorporate repeated body‐composition measures to clarify these mechanisms.

The observed inverse association between fat‐free mass and mortality likely reflects the physiologic benefits of skeletal muscle mass. Muscle tissue acts as a metabolic and endocrine organ, releasing myokines (e.g., insulin‐like growth factor‐1) with autocrine, paracrine and endocrine effects [5]. Activity‐induced myokines enhance lipid and glucose metabolism by promoting muscular glucose uptake and fat oxidation, stimulating hepatic glucose output and inducing lipolysis in white adipose tissue [38]. Collectively, these processes promote a more favourable metabolic profile, characterized by improved insulin sensitivity, greater adipose tissue oxidation, osteogenesis, anti‐inflammatory balance and antitumour effects [5]. Furthermore, higher lean mass has been shown to reduce obesity‐induced inflammation [39], thereby lowering systemic inflammation and supporting insulin sensitivity and insulin‐like growth factor‐1 levels [40].

Because many of these biological pathways are modifiable through lifestyle, residual confounding by physical activity warrants consideration. Physical activity affects body composition [41] and is associated with lower mortality [42]; however, most included studies adjusted for physical activity, supporting the robustness of our findings.

BMI may also act as a confounding variable or effect modifier. Two studies conducted analyses stratified by BMI: One found that higher mid‐arm circumference was associated with lower mortality among normal‐weight and overweight participants but not in those with obesity [S44]. Another observed higher mortality among individuals with low mid‐upper arm circumference and normal or low BMI [S33]. These findings suggest that adiposity may modify the association between arm circumference and mortality.

### 4.1 Limitations

Several limitations warrant consideration. First, between‐study heterogeneity was substantial. Although we performed meta‐regression analyses to identify potential drivers of this variance, neither publication year, sex nor measurement method significantly accounted for the observed heterogeneity. The residual heterogeneity likely reflects more complex differences in population characteristics and covariate adjustment. Variation in fat‐free mass indices and cut‐point definitions hampers comparability across studies and precludes establishing absolute thresholds for ‘low’ fat‐free mass. Method‐specific and subgroup analyses reduced—but did not eliminate—this variability; therefore, pooled estimates should be interpreted as average effects, emphasizing direction rather than magnitude. Second, adjustment strategies differed across studies, raising the possibility of residual confounding; however, sensitivity analyses, including E‐value calculations, suggest that an unmeasured confounder would need a strong association with both fat‐free mass and mortality to fully explain the observed association. Third, older adults were overrepresented in the included studies—about one‐third exclusively enrolled participants aged ≥ 65 years, whereas most others included wide age ranges without upper limits. Thus, generalizability is greatest for older populations, and more data in younger cohorts are needed. Fourth, analyses relied on baseline fat‐free mass, implicitly assuming relative stability over follow‐up. Analogous to baseline BMI, baseline measures yield risk estimates comparable to trajectory‐based approaches [43], supporting this pragmatic choice. Fifth, reverse causation remains possible. Preclinical illness may reduce fat‐free mass, inflating observed risk estimates. We mitigated this by excluding hospitalized cohorts and conducting sensitivity analyses that excluded early deaths, which modestly attenuated but did not eliminate the association. These results suggest that reverse causation contributes to, but does not fully explain, the findings. Sixth, the overall certainty of evidence according to GRADE was rated ‘low’. The evidence was downgraded due to concerns regarding risk of bias (primarily potential residual confounding) and high inconsistency arising from the diverse exposure assessment methods. Lastly, mild funnel‐plot asymmetry indicates possible publication bias, though it may also reflect heterogeneity among measurement techniques or study populations.

### 4.2 Future Directions

Given consistent evidence linking low fat‐free mass to higher mortality, measurement of fat‐free mass should become a routine component of risk appraisal alongside BMI. Pragmatic tools such as limb circumferences or BIA can be applied when standardized protocols and locally calibrated cut‐points are available, while recognizing that DXA and CT provide greater precision when accessible. Future research priorities include expanding representation of younger adults and understudied ethnic groups and employing longitudinal designs with repeated body composition measures to capture change over time. To strengthen causal inference, randomized or quasi‐experimental trials targeting fat‐free mass—through progressive resistance training, optimized protein intake or multimodal interventions—should assess effects on mortality and major cardiometabolic and cancer outcomes. These studies will clarify causal pathways, support individualized risk stratification and inform early prevention strategies.

## 5 Conclusion

This comprehensive meta‐analysis demonstrates a robust and consistent inverse association between fat‐free mass and mortality in community‐dwelling adults. Low fat‐free mass is an important determinant of higher mortality, independent of age, sex and geographic region. These findings emphasize the importance of routinely assessing body composition to identify individuals at increased risk and to guide preventive care aimed at promoting healthy longevity.

## Funding

The authors have nothing to report.

## Ethics Statement

The authors have nothing to report.

## Consent

The authors have nothing to report.

## Conflicts of Interest

The authors declare no conflicts of interest.

## Supporting information


**Table S1:** Prisma Checklist.
**Table S2:** Search terms applied to PubMed, Web of Science and EMBASE.
**Table S3:** Included studies and their characteristics.
**Table S4:** Excluded studies with continuous estimates.
**Table S5:** Overview of meta‐analysis and meta‐regressions.
**Figure S1:** Risk of bias assessment.
**Figure S2:** Analysis of publication bias: (a) funnel plot and (b) trim and fill analysis.
**Figure S3:** Bubble plot of meta‐regression for publication year.
**Figure S4:** Predicted hazard ratios for all‐cause mortality stratified by sex category.
**Figure S5:** Predicted hazard ratios for all‐cause mortality stratified by measurement device.

## Data Availability

All datasets and R code used for this meta‐analysis can be found at https://github.com/BohmannPatricia/Meta‐Analysis‐Fat‐free‐mass‐and‐Mortality.git. For additional information, please contact the corresponding author. Summary details for all included studies are available in the Supporting Information.
