# Supplementary material for: Association Between Fat‐Free Mass and Mortality: A Systematic Review and Meta‐Analysis
Source: J Cachexia Sarcopenia Muscle. 2026 Jul 10;17(4):e70331. doi: 10.1002/jcsm.70331 (PMC13353355; doi:10.1002/jcsm.70331)
Supplement: Supplementary file 1 — Table S1: Prisma Checklist. Table S2: Search terms applied to PubMed, Web of Science and EMBASE. Table S3: Included studies and their characteristics. Table S4: Excluded studies with continuous estimates. Table S5: Overview of meta‐analysis and meta‐regressions. Figure S1: Risk of bias assessment. Figure S2: Analysis of publication bias: (a) funnel plot and (b) trim and fill analysis. Figure S3: Bubble plot of meta‐regression for publication year. Figure S4: Predicted hazard ratios for all‐cause mortality stratified by sex category. Figure S5: Predicted hazard ratios for all‐cause mortality stratified by measurement device. [file JCSM-17-e70331-s001.pdf]

Supporting information: Association between fat-free mass and mortality: a systematic review and meta-analysis

Fromherz Patricia*, Haas Valerie L*, Jochem Carmen, Baurecht Hansjörg, Alt Volker, Leitzmann Michael F, Sedlmeier Anja M

**contributed equally*

Contents

Table S1 Prisma Checklist.
Table S2 Search terms applied to PubMed, Web of Science and EMBASE.
Table S3 Included studies and their characteristics.
Table S4 Excluded studies with continuous estimates.
Table S5 Overview of meta-analysis and meta-regressions.
Figure S1 Risk of bias assessment.
Figure S2 Analysis of publication bias: a) funnel plot and b) trim and fill analysis.
Figure S3 Bubble plot of meta-regression for publication year.
Figure S4 Predicted hazard ratios for all-cause mortality stratified by sex category.
Figure S5 Predicted hazard ratios for all-cause mortality stratified by measurement device.

Table S1 PRISMA Checklist.

Section and Topic	Item #	Checklist item	Location where item is reported
**TITLE**			
Title	1	Identify the report as a systematic review.	p. 1
**ABSTRACT**			
Abstract	2	See the PRISMA 2020 for Abstracts checklist.	p. 2
**INTRODUCTION**			
Rationale	3	Describe the rationale for the review in the context of existing knowledge.	p. 4
Objectives	4	Provide an explicit statement of the objective(s) or question(s) the review addresses.	p. 4-5
**METHODS**			
Eligibility criteria	5	Specify the inclusion and exclusion criteria for the review and how studies were grouped for the syntheses.	p. 5
Information sources	6	Specify all databases, registers, websites, organisations, reference lists and other sources searched or consulted to identify studies. Specify the date when each source was last searched or consulted.	p. 6
Search strategy	7	Present the full search strategies for all databases, registers and websites, including any filters and limits used.	p.6 , Table S2
Selection process	8	Specify the methods used to decide whether a study met the inclusion criteria of the review, including how many reviewers screened each record and each report retrieved, whether they worked independently, and if applicable, details of automation tools used in the process.	p. 5-6
Data collection process	9	Specify the methods used to collect data from reports, including how many reviewers collected data from each report, whether they worked independently, any processes for obtaining or confirming data from study investigators, and if applicable, details of automation tools used in the process.	p. 5-6
Data items	10a	List and define all outcomes for which data were sought. Specify whether all results that were compatible with each outcome domain in each study were sought (e.g. for all measures, time points, analyses), and if not, the methods used to decide which results to collect.	p. 6-7, Fig.1

Section and Topic	Item #	Checklist item	Location where item is reported
	10b	List and define all other variables for which data were sought (e.g. participant and intervention characteristics, funding sources). Describe any assumptions made about any missing or unclear information.	p. 5-6
Study risk of bias assessment	11	Specify the methods used to assess risk of bias in the included studies, including details of the tool(s) used, how many reviewers assessed each study and whether they worked independently, and if applicable, details of automation tools used in the process.	p. 6-7
Effect measures	12	Specify for each outcome the effect measure(s) (e.g. risk ratio, mean difference) used in the synthesis or presentation of results.	p. 6-7
Synthesis methods	13a	Describe the processes used to decide which studies were eligible for each synthesis (e.g. tabulating the study intervention characteristics and comparing against the planned groups for each synthesis (item #5)).	p. 5-6, Additional files Tables S3/S4
	13b	Describe any methods required to prepare the data for presentation or synthesis, such as handling of missing summary statistics, or data conversions.	p. 6-8
	13c	Describe any methods used to tabulate or visually display results of individual studies and syntheses.	p. 6-8
	13d	Describe any methods used to synthesize results and provide a rationale for the choice(s). If meta-analysis was performed, describe the model(s), method(s) to identify the presence and extent of statistical heterogeneity, and software package(s) used.	p. 7
	13e	Describe any methods used to explore possible causes of heterogeneity among study results (e.g. subgroup analysis, meta-regression).	p. 7-8
	13f	Describe any sensitivity analyses conducted to assess robustness of the synthesized results.	p. 7-8
Reporting bias assessment	14	Describe any methods used to assess risk of bias due to missing results in a synthesis (arising from reporting biases).	p. 7-8
Certainty assessment	15	Describe any methods used to assess certainty (or confidence) in the body of evidence for an outcome.	p. 7-8
**RESULTS**			

Section and Topic	Item #	Checklist item	Location where item is reported
Study selection	16a	Describe the results of the search and selection process, from the number of records identified in the search to the number of studies included in the review, ideally using a flow diagram.	p. 8
	16b	Cite studies that might appear to meet the inclusion criteria, but which were excluded, and explain why they were excluded.	p. 6, Fig.1, Table S4
Study characteristics	17	Cite each included study and present its characteristics.	p. 9, Table S3
Risk of bias in studies	18	Present assessments of risk of bias for each included study.	p. 9
Results of individual studies	19	For all outcomes, present, for each study: (a) summary statistics for each group (where appropriate) and (b) an effect estimate and its precision (e.g. confidence/credible interval), ideally using structured tables or plots.	Fig. 2 Table 1
Results of syntheses	20a	For each synthesis, briefly summarise the characteristics and risk of bias among contributing studies.	P. 8-10
	20b	Present results of all statistical syntheses conducted. If meta-analysis was done, present for each the summary estimate and its precision (e.g. confidence/credible interval) and measures of statistical heterogeneity. If comparing groups, describe the direction of the effect.	Table 1
	20c	Present results of all investigations of possible causes of heterogeneity among study results.	p. 9-11
	20d	Present results of all sensitivity analyses conducted to assess the robustness of the synthesized results.	p. 11-12
Reporting biases	21	Present assessments of risk of bias due to missing results (arising from reporting biases) for each synthesis assessed.	p. 11, Fig. S1
Certainty of evidence	22	Present assessments of certainty (or confidence) in the body of evidence for each outcome assessed.	p. 9-12
**DISCUSSION**			
Discussion	23a	Provide a general interpretation of the results in the context of other evidence.	p. 12
	23b	Discuss any limitations of the evidence included in the review.	p. 16-17
	23c	Discuss any limitations of the review processes used.	p. 16-17

Section and Topic	Item #	Checklist item	Location where item is reported
	23d	Discuss implications of the results for practice, policy, and future research.	p. 17
**OTHER INFORMATION**			
Registration and protocol	24a	Provide registration information for the review, including register name and registration number, or state that the review was not registered.	p. 5
	24b	Indicate where the review protocol can be accessed, or state that a protocol was not prepared.	p. 5
	24c	Describe and explain any amendments to information provided at registration or in the protocol.	p. 5
Support	25	Describe sources of financial or non-financial support for the review, and the role of the funders or sponsors in the review.	p. 19
Competing interests	26	Declare any competing interests of review authors.	p. 19
Availability of data, code and other materials	27	Report which of the following are publicly available and where they can be found: template data collection forms; data extracted from included studies; data used for all analyses; analytic code; any other materials used in the review.	p. 19

From: Page MJ, McKenzie JE, Bossuyt PM, Boutron I, Hoffmann TC, Mulrow CD, et al. The PRISMA 2020 statement: an updated guideline for reporting systematic reviews. BMJ 2021;372:n71. doi: 10.1136/bmj.n71. Page numbers refer to the submitted Main File and my change due to formatting in the publication process.

Table S2 Search terms applied to PubMed, Web of Science and EMBASE.

Set	Search Term Pub Med	Search Term Web of Science	Search Term EMBASE
Body composition (Exposition)	(“lean mass” [All fields] OR “fat free mass” [All fields] OR “fat-free mass” [All fields] OR “muscle mass” [All fields] OR “skeletal muscle mass” [All fields] OR “body composition” [All fields])	((((((((((((((ALL=(lean mass)) OR ALL=(fat free mass)) OR ALL=(fat-free mass)) OR ALL=(muscle mass)) OR ALL=(skeletal muscle mass)) OR ALL=(body composition))	('body composition'/exp OR 'lean body mass' OR 'lean mass' OR 'muscle mass' OR 'skeletal muscle mass' OR 'fat-free mass')
Mortality (Outcome)	AND (mortality [All fields] OR death [All fields])	AND ALL=(mortality OR death))	AND ('mortality'/exp OR death OR 'cause of death'/exp)
Risk estimate	AND (follow-up [All fields] OR nested [All fields] OR “relative risk” [All fields] OR “hazard ratio” [All fields] OR “odds ratio” [All fields] OR cohorts [All fields])	AND ALL=(follow-up OR nested OR relative risk OR hazard ratio OR odds ratio OR cohorts))	AND ('relative risk' OR 'hazard ratio' OR 'odds ratio' OR 'follow-up')
Limit: Publication type included	NOT (editorial[ptyp] OR comment[ptyp] OR letter[ptyp] OR guideline[ptyp] OR news[ptyp])	NOT DT=(Editorial Material OR Letter OR News Item))	full text and human and "remove medline records" and (english or german) and (article or article in press or conference paper or data paper or "preprint (unpublished, non-peer reviewed)" or short survey)
Limit: Exclude treatment	NOT (treatment[tiab] OR therapy[tiab] OR sarcop*[title])	NOT TI=(treatment OR therapy OR sarcop*)) NOT AB=(treatment OR therapy OR sarcop*))	
Limit: Exclude child only | include human adults	NOT ((“infant”[Mesh] OR “child”[mesh] OR “adolescent”[mh]) NOT ((“infant”[Mesh] OR “child”[mesh] OR “adolescent”[mh]) AND “adult”[Mesh] AND humans[Mesh]))	NOT AK=(infant OR child OR adolescent)))))	

Table S3 Included studies and their characteristics.

Author, year, country [reference]	Study name	Follow-up duration (years)	Men, women, combined	Number of participants / deaths	Age range in years	Exposition: body composition/ measurement method	Outcome: mortality assessment	CVD[1] mortality definition	Cancer mortality definition	Risk estimate (HR, OR)[2]	Adjustment variables
Kim D, 2024, USA [S19]	NHANES 1999-2006, 2011-2018	1999/2006 -12/2019 2011/2018 - 12/2019 NA	Men and women combined	16839/ 2109	>20	Appendicular skeletal muscle index in kg/m² by DXA	National death index	NA	-	HR	Age, sex, race, smoking status, alcohol consumption, estimated glomerular filtrtion rate, central obesity, history of cancer
Liu CA, 2023, USA [S1]	NHANES 2003-2006	2003-06 - 2019, Mean 14.625 years	Men and women combined	5052/826	20-59	Total lean mass in g by DXA	National death index	-	-	HR	Age, sex, race/ethnicity , education level, marital status, family income-poverty ratio level, hypertension, coronary heart disease, diabetes,

[1] CVD: Cardiovascular disease
[2] HR: Hazard ratio, OR: Odds ratio

											cancer, smoke, covered by health insurance, alcohol, BMI, waist, muscle strengthenin g activities, Healthy Diet Index score
Bernabe-Ortiz A, 2023, Peru [S46]	CRON ICAS cohort study	2010-2018, Mean 7.0 years	Men and women combined	3216/ 172	≥30	Skeletal muscle mass in kg by BIA	National records	-	-	HR	Age, sex, education level, socioeconom ic level, daily smoking, alcohol use, physical activity levels and body mass index
Chang CS, 2023, Taiwan [S25]	Cohort from Towns hips in Taiwa n	2009/10-2018	Men	224/ NA	≥65	Skeletal muscle mass index in kg/m² by BIA	Taiwan cause of death database	-	-	HR	Exercise, gender, age, socioeconom ic status, smoking, alcohol consumption, and Charlson comorbidity index
Liu J, 2023, US [S23]	NHAN ES	1999-2006, Mean 11.8 years	Men and women	17735/ 3446	≥65	Anthropometric circumference measurements in cm	Interviews with close family members	ICD-10: 100-178	-	HR	Age, sex, race/ethnicity , education

Ying Z, 2023, Taiwan [S26]	Taiwa n MJ Cohort	1997-2011, Mean 9 years	Men and women combined	422230/ 11892	≥20	Fat-free mass index in kg/m² by BIA	Linkage of health data to mortality data	ICD-9 and ICD-10, no further specificatio n	ICD-9 and ICD-10, no further specificatio n	HR	Fat mass index, age, gender, marital status, education, occupation, smoking status, drinking status, physical activity, and dietary intake (light vegetable, dark vegetable, fruit, and meat intake)
Camargo Pereira C, 2022 [S20]	Goiâni a Older Adults project	2008-2018/19, Mean 10 years	Men and women combined	418/ 147	≥60	Arm muscle circumference in cm²	Brazilian Mortality Information System	-	-	HR	Age, sex, skin colour, education, socioeconom ic class, marital status,age, smoking, alcohol consumption, physical activity, consumption of fruits and vegetables
Landi F, 2022, Italy [S21]	IISIRE NTE study	2003/04-2013/14, NA	Men and women combined	346/ 245	≥80	Appendicular skeletal muscle in kg	National Death registry	-	-	HR	Age, gender, ADL (activities of daily living)

											impairment, cognitive impairment, BMI (body mass index), CRP (C-reactive protein), and IL- 6 (interleukin 6)
Li C, 2022, Taiwan [S22]	Taichung, Taiwa n community-based cohort study	2009-2021, Mean 12 years	Men and women combined	641/ 198	≥65	Skeletal muscle index in kg/m² by DXA	National Registry of Death	-	-	HR	Age, sex, education, marital status, smoking, alcohol drinking, physical activity, exercising program, hypertension, diabetes mellitus, heart disease, stroke, cancer, cognitive impairment, fasting
Tabara Y, 2022, Japan [S47]	Nagahama study	2008/10-2013/16 and 2022, Mean 7.02 years	Men and women	3582/ 189	≥65	Skeletal muscle index in kg/m² by BIA	Reviewing residential registry records	-	-	HR	Age, BMI, history of cardiovascular disease, history of cancer, current

											smoking, systolic blood pressure, albumin, haemoglobin A1c, high-density lipoprotein cholesterol, low-density lipoprotein cholesterol, C-reactive protein
Wu M, 2022, China [S24]	China Kadoorie Biobank study	2004/08-2017,2013/ 14-2017 , Mean 3.98 years	Men and women	23290/ 739	38-88	Appendicular muscle mass index in kg/m² by BIA	Disease Surveillance Points in China	-	-	HR	Sex, educational attainment, marital status, occupation, household income, smoking status, alcohol consumption, levels of physical activities, scores of dietary patterns, prevalent hypertension, prevalent diabetes, prevalent COPD

Liu M, 2022, US [S11]	NHANES, NHANES III	1988/1994-2015; 1999/2014-2015 Median 9.7 years	Men and women combined	55818/ 10408	≥18	Predicted lean mass in kg by anthropometric prediction equations	Linkage to the National Death Index	ICD 10: I00-09, I11, I13, I20-25, I26-51, I60-I69	ICD 10: C00-C97	HR	Age, sex, height, race/ethnicity, education level, marital status, smoking status, history of hypertension and diabetes, leisure physical activity level, HDL cholesterol, total cholesterol, predicted fat mass
Knowles R, 2021, UK [S48]	UK Biobank	2006/2010-2020 Median 10.5 years	Men, women separately	356590/ 15844	40-69	Appendicular skeletal muscle mass in kg by BIA	Linkage to the National Death Index	-	-	HR	Age, height, Townsend deprivation index, education, smoking, alcohol intake, physical activity, oily fish intake, fruit and vegetable intake, saturated fat intake, diabetes, cancer history,

											menopause (women)
Sedlmeier AM, 2021, Germany, US [S14]	Poole d study of KORA , SHIP and NHAN ES	1994/1994 -2016; 1999/2001 -2016; 1999/2000 -2015; 2001/2002 -2015; 2003/2004 -2015; 2008-2015 Median 14.3 years	Men and women combined	16155/ 1347	25-74, 20-49, 20-79	Fat-free mass index in kg/m² by BIA	Population registries and death certificates from local health authorities, linkage to the National Death Index	-	-	HR	Age, sex, cohort, ethnicity, baseline history of diabetes, education, smoking, physical activity, alcohol intake, fat mass index
Cawthon P, 2021, US [S32]	MrOS (Osteo porotic fractur es in men) study	2014/16-2019 Mean 3.3 years	Men	1400/ 197	77-101	Appendicular lean mass in kg by DXA	Notification during follow-up (every 4 months), death certificates	ICD-9: 401.90 to 442.0, 394.0, 394.9, 396.9, 398.9, 401.1, 443.9, 459.7, 459.9, 557.0, 785.51, 996.71	ICD-9: 140.0–208.0	HR	Age, ethnicity, clinical center, alcohol, smoking, comorbidities , physical activity, percent fat, exhaustion, cognitive function, self-reported health status, weight change, weight, height, strength & physical performance

											(chair stand, gait speed and grip strength)
He L, 2021, China [S8]	The China Health and Nutriti on Study (CHN S)	1993-2015<br><br>Median 9.2 years	Men and women combined	17717/ 167	≥18	Mid-upper arm circumference in cm	Report from family members	-	-	HR	Age, sex, BMI[3], marital status, education, income, region, urbanization index, smoking, alcohol, physical activity, total energy intake, fat intake, protein intake, carbohydrate intake, blood pressure (systolic, diastolic), diabetes, TSF[4] thickness, subcutaneou s fat
Fernandes DPD, 2021, Brazil [S6]	Popul ation from the	2009-2018<br><br>NA	Men and women combined	796/ 197	≥60	Calf circumference in cm	Mortality Information System by Viscosa	-	-	HR	Age, sex, education, quality of diet, physical

[3] BMI: Body Mass Index
[4] TSF: triceps skinfold

	National Older Adults Vaccination Campaign from a Brazilian City						Municipal Health Department				activity, smoking, BMI
Soerensen TIA, 2020, Denmark [S16]	Danish MONICA project	1993/94-2012 NA	Men and women combined	1951/ 486	35-65	Fat-free muscle mass index in kg/m² by BIA	Linkage to the national Central Person Register	-	-	HR	Age, sex, smoking, alcohol intake, physical activity, education
Seino S, 2020, Japan [S15]	Kusatsu Longitudinal Study and Hatoyama Cohort Study	2008/2016-2017; 2010/2014-2015 Median 5.3 years	Men, women separately	966/ 128 (men) 1011 /75 (women)	65-74; ≥75	Fat-free muscle mass index in kg/m² by BIA	Linking records with the Japanese National Vital Statistics	-	-	HR	Age, FM[5], study area, year, alcohol, smoking, hypertension, CVD[6] (stroke, heart disease), diabetes, cancer, cholesterol level, hypoalbumin

[5] FM: Fat mass
[6] CVD: Cardiovascular disease

											emia, anemia, chronic kidney disease
Costanzo L, 2020, Italy [S35]	InCHI ANTI study	2001-2010 Mean 3.1	Men and women combined	535/ 56	≥65	Skeletal muscle index in kg/m² by BIA	Mortality General Registry by the Tuscany Region and from death certificates of the municipality of residence	-	-	HR	Age, sex, BMI[1], marital status, education, and comorbidities
Oh H, 2020, Korea [S41]	Korea Nation al Health and Nutriti on Exami nation Surve y 2007–2018	2008/2011 -2016 NA	Men and women combined	17284/ 1072	20-95	Skeletal muscle index in kg/m² by DXA	Matching death certificates and medical records based on the resident registration number	ICD-10: I00–I99	ICD-10: C00–D48	HR	Marital status, residence, income, occupation, smoking, alcohol, predicted fat mass, height
Larsen B, 2020, US [S37]	Muli-Ethnic Study of Athero sclero sis (MES A)	2002-NA Mean 10.6 years (men) 10.9 years (women)	Men, women separately	946/118 (men) 955/119 (women)	45-85	Abdominal muscle area in cm²/m² by CT	Phone calls and death certificates	-	-	HR	Age, ethnicity, height, diabetes, systolic blood pressure, CVD[4] medication (antihyperten sive

											medication, statin use), total cholesterol, HDL cholesterol, smoking, cancer, kidney function, physical activity, sedentary time, visceral fat, BMI[1]
De Almeida Roediger M, 2019, Brazil [S2]	SABE (Health, Well-being, and Aging)	2000-2010 NA	Men and women combined	1504/ 769	≥60	Mid-upper arm circumference in cm<br><br>Calf circumference in cm	Fundação Sistema Estadual de Análise de Dados; PRO-AIM - Programa de Aprimorament o das Informações de Mortalidade	-	-	HR	Age, sex, marital status, education, working status, income, alcohol, physical activity, smoking, hypertension, CVD[4], lung disease, stroke, cancer, number of diseases, MMSE[7], geriatric

[7] MMSE: Mini Mental State exam

											depression scale
Wang H, 2019, China [S42]	Projec t of Longe vity and Aging in Dujian gyan (PLAD)	2005-2009 Median 4 years	Men, women separately	238/ 132 (men) 500/ 255 (women)	90-105	Skeletal muscle index in kg /m² by anthropometric equations	Requested from local government registries and confirmed by relatives or neighbors	-	-	HR	Age, smoking, alcohol, cognitive impairment (MMSE[5]), disability
Loprinzi PD, 2018, US [S40]	Nation al Health and Nutriti on Exami nation Surve y (NHA NES)	1999/2002 -2011 Median 10.3 years	Men and women combined	1079/ 277	50-85	Leg lean mass in g by DXA	Linkage to the National Death Index	-	-	HR	Age, sex, race-ethnicity, relative protein intake, carbohydrate intake, fat intake, mean arterial pressure, smoking status
Li R, 2018, US [S39]	Nation al Health and Nutriti on Exami nation Surve	1999/2002 -2011	Men and women combined	4449/ NA	≥50	Appendicular lean mass in kg by DXA	Public linked mortality files, death certificates	-	-	OR	Age, sex, ethnicity, BMI[1], smoking, alcohol, education, physical activity (LTPA[8]),

[8] LTPA: leisure time physical activity

	y (NHA NES)										sedentary time, cardiovascul ar disease, diabetes, cancer, chronic obstructive lung disease, chronic kidney disease
Lee DH, 2018, US [S38]	The Health Profes sional s Follow -up Study	1987-2012; 1996-2012; 2008-2012 Mean 21.4 years	Men	38006/ 12356	40-75	Predicted lean mass in kg by anthropometric prediction equations	Reports from the next of kin or postal authorities or by searching the National Death Index	ICD-8: 390-459	ICD-8: 140-239	HR	Age, ethnicity, family history of CVD[4], family history of cancer, physical activity, alcohol consumption, total energy intake, smoking, Alternate Healthy Eating index
Batsis JA, 2017, US [S3]	Nation al Health and Nutriti on Exami nation Surve y	1999/2004 -2016 Median 8.5 years	Men and women combined	4984/ 1901	≥60	Appendicular lean mass in kg by DXA	Linkage to National Death Index	ICD-10: I00-I78	-	HR	Age, sex, ethnicity, poverty income ratio, smoking, diabetes, CVD[4] (congestive heart failure, coronary heart

	(NHANES)										disease), non-melanoma skin cancer, arthritis, physical activity
Pasco JA, 2017, Australia [S13]	The Geelong Osteoporosis Study	1993/1997 - 2003/2007 NA	Women	750/ 190	50-92	Appendicular lean mass in as t-scores by DXA	Linkage to the Australian National Deaths Index	-	-	HR	Age, weight, height, BMI[1], mobility, polypharmac y
Balogun S, 2017, Australia [S29]	Tasmanian Older Adult Cohort (TASOAC)	2002/2004 -2015; 2005/2006 -2015; 2007/2009 -2015 NA	Men and women combined	1041/ 145	51-81	Appendicular lean mass index in kg/m² by DXA	National and state death registry	-	-	HR	Age
Wu L, 2017, US [S44]	The Third National Health and Nutrition Examination Surve y (NHA	1988/1994 -2006 Median 14.3 years	Men and women combined	11958/ NA	20-90	Mid-arm circumference in cm	Probabilistic matching between National Health Index certificate records and NHANES III participants	NA	-	HR	Age, sex, ethnicity, BMI[1], serum triglycerides, serum aspartate transaminase , serum HDL, serum glucose, CRP[9], serum uric acid, serum total bilirubin, systolic blood

[9] CRP: C-reactive protein

	NES III)										pressure, smoking, diabetes, physical activity
Wu L, 2017, US [S49]	The Third National Health and Nutrition Examination Surve y (NHANES III)	1988/1994 -2006 Median 14.3 years	Men, women separately	3373/ 1396 (men) 3396/ 1097 (women)	40-90	Mid-arm muscle circumference in cm	Probabilistic matching between National Health Index certificate records and NHANES III participants	NA	-	HR	Age, ethnicity, BMI[1], waist circumference, serum total cholesterol, serum HDL, serum glucose, CRP[7], serum uric acid, serum total bilirubin, systolic blood pressure, smoking, diabetes, congestive heart failure, serum albumin, marital status, number of prescription medications taken
Cheung CL, 2016, US [S34]	National Health and Nutriti	1999/2004 -2006 NA	Men and women combined	2304/ 330	≥65	Appendicular lean mass index in kg/m² by DXA	Linkage to the National Death Index	-	-	HR	Age, sex, ethnicity, smoking, alcohol, CRP[7], triglycerides,

	on Exami nation Surve y (NHA NES)										HDL cholesterol, eGFR[10]
Zong G, 2016, US [S45]	Nation al Health and Nutriti on Exami nation Surve y (NHA NES)	1999/2006 -2010 Mean 8.8 years	Men and women combined	9471/ 682	≥20	Fat-free mass index in kg/m² by DXA	Linkage to the National Death Index	ICD-10: I00-I79	ICD-10 C00-C97	HR	Age, sex, ethnicity, education, marital status, family income to poverty ratio, family history of chronic diseases (diabetes, hypertension, stroke, or angina) physical activity, smoking status, alcohol consumption, BMI[1]
Bea JW, 2015, US [S31]	WHI Clinica l Trials and Obser vation al Study	1993/1998 - 2007 Mean 13.6 years	Women	10525/ 1762	50–79	Lean mass in % by DXA	Medical records and death certificates, National Health Index	-	-	HR	Age, age at menopause, physical activity, diet quality, ethnicity, smoking,

[10] eGFR: estimated Glomerular Filtration Rate

											alcohol, hormone use
Srikanthan P, 2014, US [S17]	The Third National Health and Nutrition Examination Surve y (NHANES III)	1988/1994 -2004 Median 13.2 years	Men and women combined	3659/ 2012	≥55	Skeletal muscle mass index in kg/m² by BIA	National Death Index record, Social Security Administration, Centers for Medicare and Medicaid Services	-	-	HR	Age, sex, ethnicity, central obesity, smoking, cancer, CRP[7], hypertension, cholesterol (HDL and total), HOMA-IR, HbA1C, diabetes, prediabetes, serum creatinine
Chuang SY, 2014, Taiwan [S5]	The Elderl y Nutrition and Health Surve y in Taiwan (1999-2000) (Elderly NAHSIT)	1999/2000 -2008 Median 9.2 years	Men and women combined	1512/ 506	≥65	Skeletal muscle mass index in kg/m² by BIA	National Death Registry through personal identification number	ICD-9: 390-459	-	HR	Age, sex, BMI, smoking, alcohol, physical activity, CRP[7], eGFR[8], number of comorbidities

Batsis JA, 2014, US [S30]	The Third National Health and Nutrition Examination Surve y (NHANES III)	1988/1994 -2006 Mean 11.9 years	Men and women combined	1569/ 792	≥60	Lean mass in kg by BIA	Linkage to National Death Index	ICD-10: I00-I78	-	HR	Age, sex, ethnicity, smoking, CVD[4], self-reported health, hypertension, diabetes, dyslipidemia, physical activity
Chen Y, 2014, Bangladesh [S33]	Health Effect s of Arsenic Longitudinal Study (HEALS)	2000/2002 -2012; 2006/2008 -2012 Mean 7.9 years	Men and women combined	19575/ 744	≥18	Mid-upper arm circumference in cm	Report by family or neighbours, death certificates	ICD-10: I00-I99	ICD-10: C00-C97	HR	Age, sex, BMI[1], education level, betel use, smoking status, baseline systolic blood pressure
Genton L, 2013, Switzerland [S7]	Geneva university hospital - recruitment of health	1999-2010; 2002-2010; 2005-2010; 2008-2010 NA	Men and women combined	203/ 58	≥65	Fat-free mass index in kg/m² by BIA	Investigators checked with the administration of the cities they were living in at the last follow-up	-	-	HR	Age, sex, FMI[11], physical activity, Charlson index

[11] FMI: Fat mass index

	y subjec ts throug h adverti semen t										
Bites AC, 2013, Chile [S4]	Insitiut o de Nutrici on y Tecnol ogia de Alime ntos (INTA) Univer sity of Chile	1999/2005 -2008 Median 7.2 years	Men	75/ 23	61-91	Lean mass/height in g/cm by DXA	Mortality records of Sevicio de Registro Civil e Identificacion del Ministerio de Justica del Gobierno de Chile	-	-	HR	Age, hand grip strength, 12-min walking capacity, total lean mass/height²
Tsai AC, 2011, Taiwan [S18]	Surve y of Health and Nutriti on Status of the Elderl y in Taiwa n	1999-2003 NA	Men and women combined	4191/ 566	≥53	Mid-arm circumference in cm<br><br>Calf circumference in cm	Records from Universal Health Insurance Program and the National Household Registration	-	-	HR	Age, sex, smoking, physical activity
Landi F, 2010, Italy [S10]	Aging and Longe vity	2002/2003 - 2007/2008	Men and women combined	357/ 146	≥80	Mid-arm muscle circumference in cm	National Death Registry	-	-	HR	Age, sex, living alone, sensory impairments,

	Study in the Sirente Geographic Area (ilSIRENTE Study)										albumin, cholesterol, body mass index
Heitmann BL, 2009, Denmark [S9]	The Danish MONICA project	1987-2002 Mean 12.5 years	Men	1436/ 257	35-65	Thigh circumference in cm	Personal identification number at National Registers of Hospital Discharge and Death Registry	-	-	HR	Age, smoking, physical activity, education, body fat percentage, body height, BMI[1], waist circumference, alcohol, systolic blood pressure, total cholesterol, triglycerides
Wannamethee SG, 2007, UK [S43]	The British Regional Heart Study	1998/2000 -2005 Mean 6.0 years	Men	4107/ 713	60–79	Fat-free mass index in kg/m² by BIA	Established “tagging” procedures provided by the National Health Service registers	-	-	HR	Age, social class, physical activity, alcohol intake, smoking
Dolan CM, 2007, US [S36]	The Study of Osteo	1989/1991 -1997 Mean 8.0 years	Women	8029/ 945	≥65	Lean mass in kg by BIA	Study participants were contacted	-	-	HR	Age, self-reported health, grip strength,

	porotic Fractures						every 4 months				non-thiazide diuretic use, femoral neck bone mineral density
Miller MD, 2002, Australia [S12]	Australian Longitudinal Study of Ageing (ALSA)	1992-2000 NA	Men and women combined	1396/ 579	≥70	Corrected arm muscle area in cm²	Every 12 months from government birth, death and marriage records	-	-	HR	Age, sex, marital status, smoking, self-rated health, activities of daily living, cancer, CVD[4], diabetes, hypertension, respiratory disease, depression (CESD[12]), cognitive impairment (MMSE[5])
Moon S, 2025, Korea & US [S27]	National Health and Nutrition Examination Survey (NHANES)	NHANES: 1999-2006, 2011-2018 & KNHANES 2008-2011 till December 2019	Men and women combined	NHANES: 8036/1058<br><br>KNHANES: 14449/1045	NHANES: 48.0±17.1<br><br>KNHANES: 48.5±16.4	Appendicular skeletal mass index in kg/m^2 by DXA	Linkage to mortality data at the National Center for Health Statistics & National Death Registry of the Korean National	-	-	HR	Age, sex, ethnicity, smoking status, alcohol consumption, history of cancer, estimated glomerular filtration rate, dyslipidemia

[12] CESD: Center Epidemiological Studies Depression scale

	& Korea National Health and Nutrition Examination Surve y (KNHANES)						Statistical Office				and HTN at baseline
Cheng Y, 2025, US [S28]	National Health and Nutrition Examination Surve y (NHANES)	1999-2006, 2011-2018 till December 2018; 10.9 year median follow-up	Men and women combined	21938/2885	20-85	Appendicular skeletal muscle mass index in kg/m^2 by DXA	Linkage to the National Death Index	-	-	HR	Age, sex, ethnicity, marital status, educational level, family income-to-poverty ratio, BMI, history of chronic diseases

Table S4 Excluded studies with continuous estimates.

Author	Year	Body composition measure	Type device	Unit	Continuous estimate
Sun Z	2024	Calf circumference	Anthropometric measurement	cm	
Wang Y	2024	ASMI	DXA	kg/m²	
Orwoll E	2021	D3Cr muscle mass	D3Cr	kg	per 1 unit increase
Howell CR	2018	Lean mass	BIA	kg	per 5 kg
Bigaard J	2005	FFMI	BIA	kg/m²	
Abramowitz MK	2018	ASMI	DXA	kg/m²	per 1 kg/m²
Spahillari A	2016	Total lean mass	DXA	kg /m²	per 1 kg/m²
Kim YH	2016	ASMI	DXA	kg/m²	
Navaneethan SD	2014	Lean mass	DXA	kg	per 10 kg increase
Wijnhoven HAH	2012	ASMM	DXA	kg	per 1 SD lower value
Farsijani S	2021	Muscle area	CT	cm²	per SD
Newman AB	2006	CT leg muscle area	CT	cm²	per 28,1 cm²
Wijnhoven HAH	2010	Mid-upper arm circumference	MUAC	cm	per 1 SD decrease
Mason C	2008	Arm circumference	AC	cm	per SD
Zhu S	2003	Upper arm circumference	MUAC	mm	
Allison DB	2002	Upper arm circumference	MUAC	cm	
Kawamoto R	2021	Thigh circumference	TC	cm	per 1 cm increment
Lee DH	2018	(Predicted) lean body mass	APE	kg	per SD
Gale CR	2007	Fat-free mass	APE	kg	per SD increase
Metter EJ	2002	Total body muscle mass	24 h creatinine excretion	g/24h	per SD

FFMI: fat-free mass index, ASMI: appendicular skeletal muscle index, ASMM: appendicular skeletal muscle mass, D3Cr: D3-Creatine dilution method, BIA: Bio-impedance analysis, DXA: dual energy X-ray absorptiometry, CT: computer tomography, AC: arm circumference, MUAC: mid-upper arm circumference, TC: thigh circumference, APE: anthropometric prediction equation

Table S5 Overview of meta-analysis and meta-regressions.

**model**	**Moderator**	**studies**	**Estimate (95% CI)**	**p-value**	**Heterogeneity (I^2)**	**Explained Variance (R^2)**
Main analysis	Overall effect (Random-effects model)	48	1.42 (1.30-1.55)	<.001	86.2%	-
Meta-regression (univariate)	Publication year (continuous)	48	β = 0.01	0.208	85.2%	6.4%
Meta-regression (univariate)	Sex	48		0.189	83.1%	9.9%
Meta-regression (univariate)	Assessment method	47		0.132	83.0%	10.2%
Meta-regression (multivariate)	Publication year + sex + assessment method	47		0.116	79.9%	18.7%

Figure S1 Risk of bias assessment.

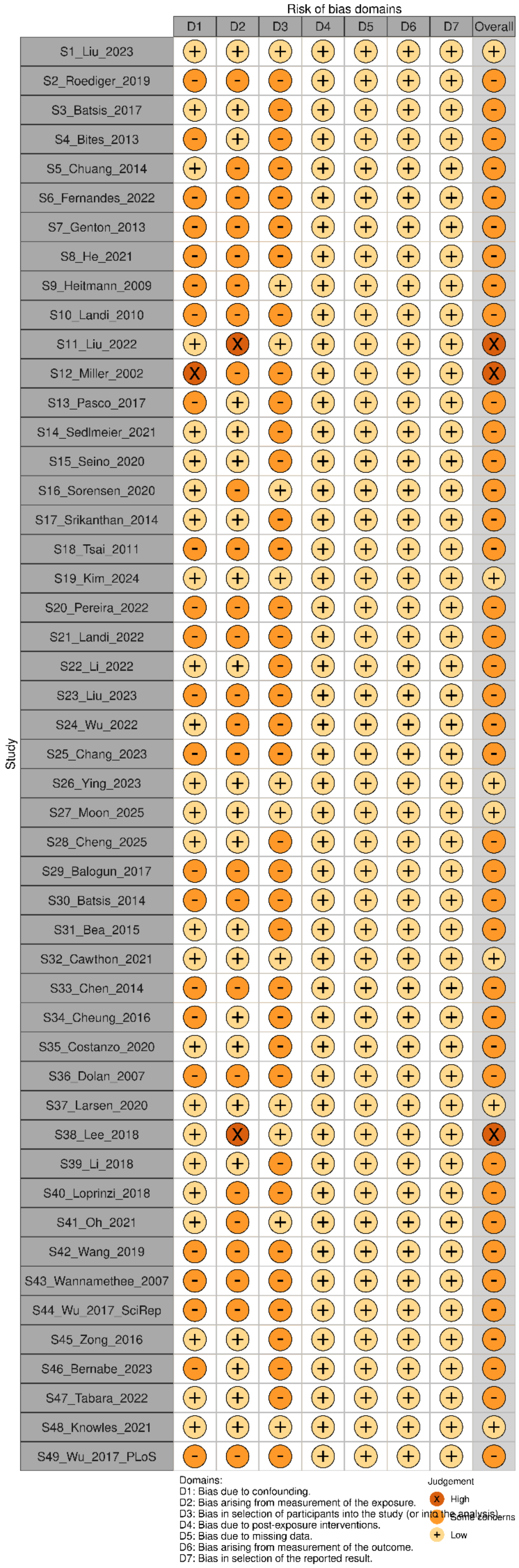

Figure S2 Analysis of publication bias: a) funnel plot and b) trim and fill analysis.

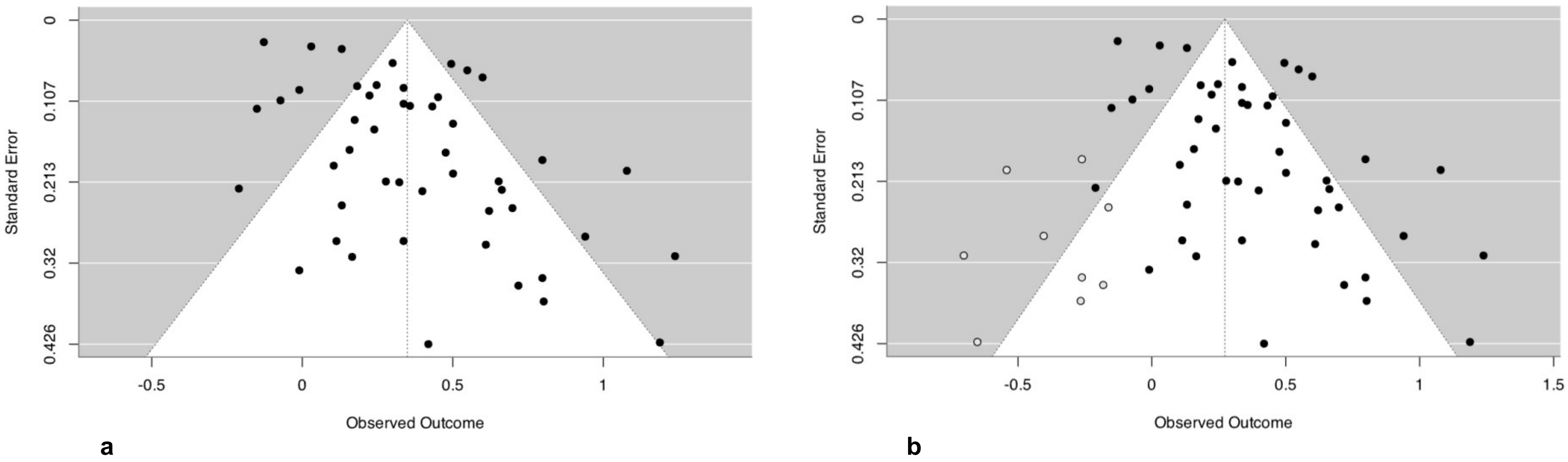

Figure S3 Bubble plot of meta-regression for publication year.

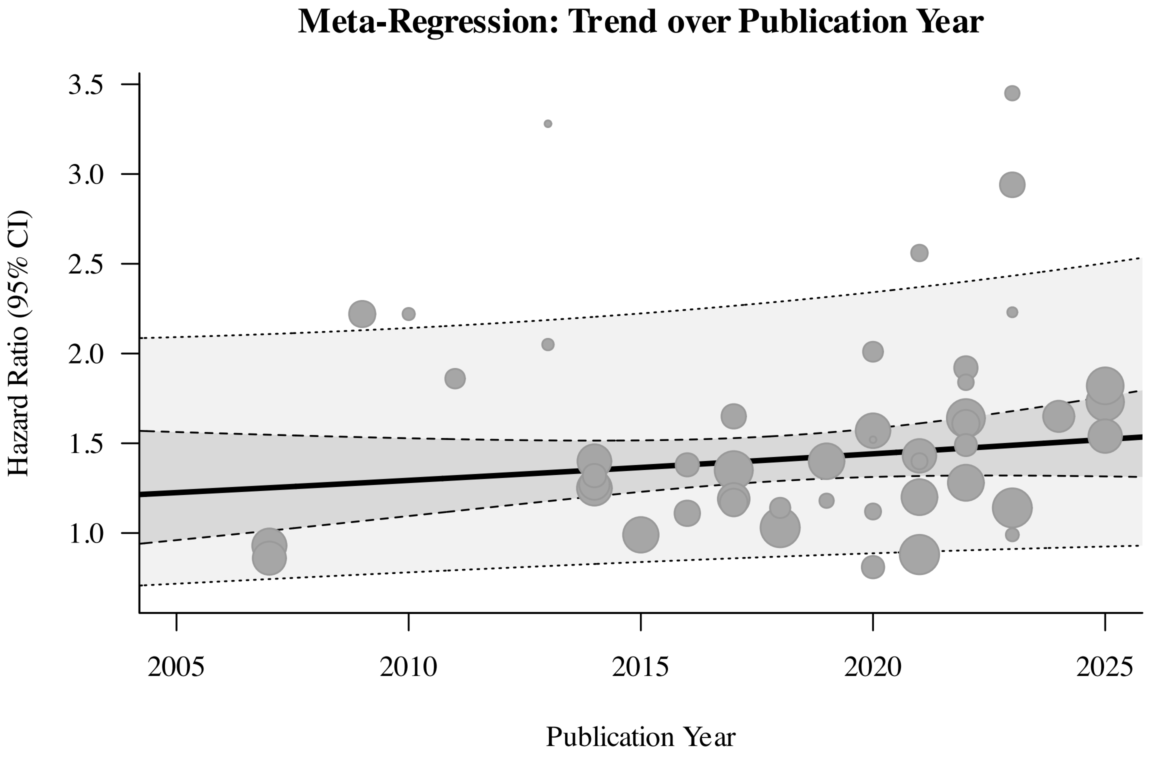

Note: The plot shows the relationship between publication year and the hazard ratio (HR) for all-cause mortality associated with low fat-free mass. Bubble sizes reflect the precision (inverse variance of each study). No significant temporal trend was observed.

Figure S4 Predicted hazard ratios for all-cause mortality stratified by sex category.

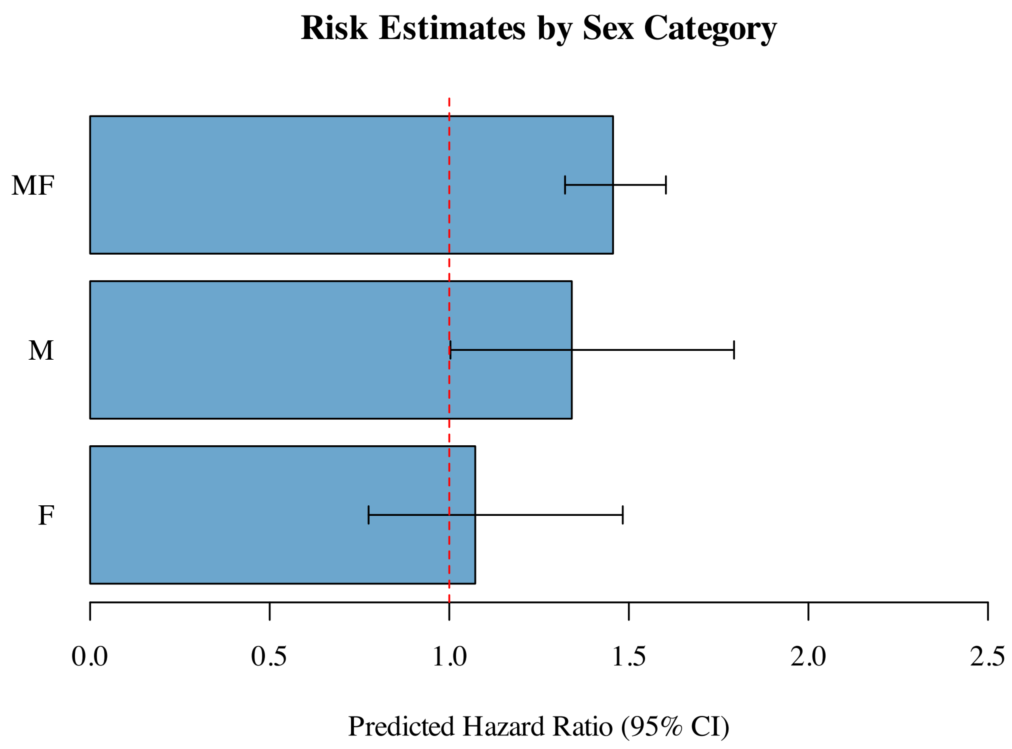

Note: Bars represent the predicted hazard ratios (HR) derived from the categorical meta-regression model, with error bars indicating the 95% confidence intervals. The vertical red dashed line represents the null effect (HR = 1.0). F: female cohorts; M: male cohorts; MF: mixed cohorts. The overall test for the moderator 'sex' was not statistically significant.

Figure S5 Predicted hazard ratios for all-cause mortality stratified by measurement device.

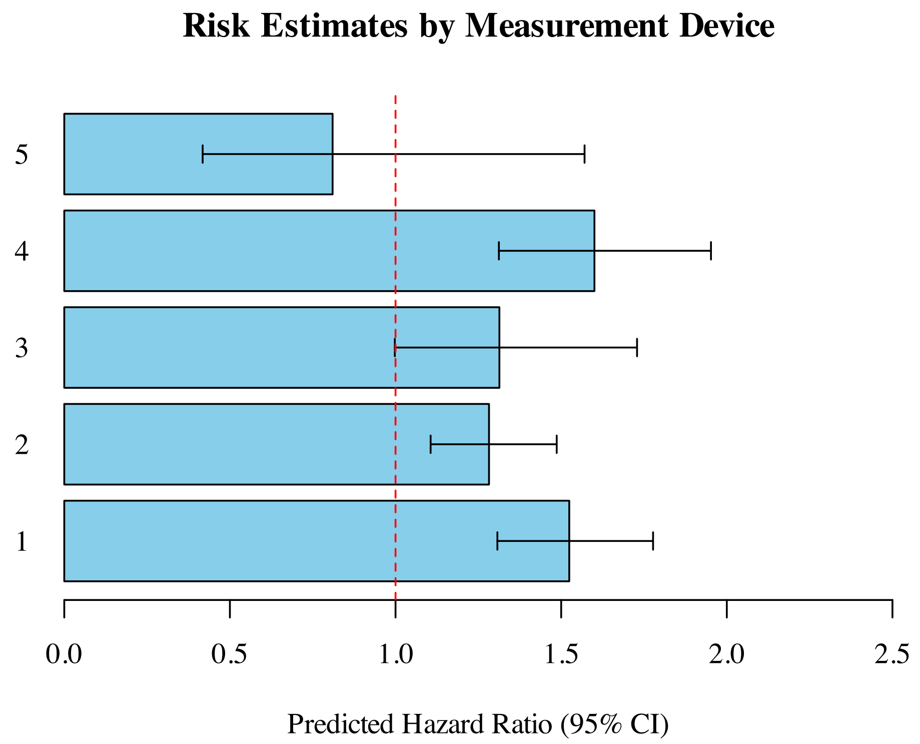

Note: Bars represent the predicted hazard ratios (HR) derived from the categorical meta-regression model, with error bars indicating the 95% confidence intervals. The vertical red dashed line represents the null effect (HR = 1.0). Numbers 1 to 5 correspond to the measurement methods (1: DXA, 2: BIA, 3: predictive equations, 4: circumference measures, 5: CT). The overall test for the moderator 'measurement device' was not statistically significant.

Supporting References from the Manuscript

[S1] C.-A. Liu *et al.*, 'Muscle distribution in relation to all-cause and cause-specific mortality in young and middle-aged adults', *Journal of Translational Medicine*, vol. 21, no. 1, p. 154, Feb. 2023, doi: 10.1186/s12967-023-04008-7.

[S2] M. de Almeida Roediger, M. de Fátima Nunes Marucci, D. A. Quintiliano Scarpelli Dourado, C. de Oliveira, J. Licio Ferreira Santos, and Y. A. de Oliveira Duarte, 'Body Composition Changes and 10-Year Mortality Risk in Older Brazilian Adults: Analysis of Prospective Data from the SABE Study', *J Nutr Health Aging*, vol. 23, no. 1, pp. 51–59, 2019, doi: 10.1007/s12603-018-1118-1.

[S3] J. A. Batsis, T. A. Mackenzie, R. T. Emeny, F. Lopez-Jimenez, and S. J. Bartels, 'Low Lean Mass With and Without Obesity, and Mortality: Results From the 1999-2004 National Health and Nutrition Examination Survey', *J Gerontol A Biol Sci Med Sci*, vol. 72, no. 10, pp. 1445–1451, Oct. 2017, doi: 10.1093/gerona/glx002.

[S4] A. C. Bites, D. Bunout, G. Barrera, S. Hirsch, L. Leiva, and M. P. De La Maza, 'Association Between Functional Measures and Mortality in Older Persons', *International Journal of Gerontology*, vol. 7, no. 1, pp. 17–21, Mar. 2013, doi: 10.1016/j.ijge.2012.05.005.

[S5] S.-Y. Chuang, H.-Y. Chang, M.-S. Lee, R. Chia-Yu Chen, and W.-H. Pan, 'Skeletal muscle mass and risk of death in an elderly population', *Nutr Metab Cardiovasc Dis*, vol. 24, no. 7, pp. 784–791, Jul. 2014, doi: 10.1016/j.numecd.2013.11.010.

[S6] D. P. de Souza Fernandes, L. L. Juvanhol, M. Lozano, and A. Q. Ribeiro, 'Calf circumference is an independent predictor of mortality in older adults: An approach with generalized additive models', *Nutr Clin Pract*, vol. 37, no. 5, pp. 1190–1198, Oct. 2022, doi: 10.1002/ncp.10780.

[S7] L. Genton, C. E. Graf, V. L. Karsegard, U. G. Kyle, and C. Pichard, 'Low fat-free mass as a marker of mortality in community-dwelling healthy elderly subjects', *Age Ageing*, vol. 42, no. 1, pp. 33–39, Jan. 2013, doi: 10.1093/ageing/afs091.

[S8] L. He *et al.*, 'Mid-Arm Muscle and Subcutaneous Fat Associated with All-Cause Mortality Independent of BMI: A Prospective Cohort Study', *Obesity (Silver Spring)*, vol. 29, no. 7, pp. 1203–1214, Jul. 2021, doi: 10.1002/oby.23179.

[S9] B. L. Heitmann and P. Frederiksen, 'Thigh circumference and risk of heart disease and premature death: prospective cohort study', *BMJ*, vol. 339, p. b3292, Sep. 2009, doi: 10.1136/bmj.b3292.

[S10] F. Landi *et al.*, 'Midarm muscle circumference, physical performance and mortality: results from the aging and longevity study in the Sirente geographic area (ilSIRENTE study)', *Clin Nutr*, vol. 29, no. 4, pp. 441–447, Aug. 2010, doi: 10.1016/j.clnu.2009.12.006.

[S11] M. Liu *et al.*, 'Predicted fat mass and lean mass in relation to all-cause and cause-specific mortality', *J Cachexia Sarcopenia Muscle*, vol. 13, no. 2, pp. 1064–1075, Apr. 2022, doi: 10.1002/jcsm.12921.

[S12] M. D. Miller *et al.*, 'Corrected arm muscle area: an independent predictor of long-term mortality in community-dwelling older adults?', *J Am Geriatr Soc*, vol. 50, no. 7, pp. 1272–1277, Jul. 2002, doi: 10.1046/j.1532-5415.2002.50316.x.

[S13] J. A. Pasco, M. Mohebbi, K. L. Holloway, S. L. Brennan-Olsen, N. K. Hyde, and M. A. Kotowicz, 'Musculoskeletal decline and mortality: prospective data from the Geelong Osteoporosis Study', *J Cachexia Sarcopenia Muscle*, vol. 8, no.

3, pp. 482–489, Jun. 2017, doi: 10.1002/jcsm.12177.
[S14] A. M. Sedlmeier *et al.*, 'Relation of body fat mass and fat-free mass to total mortality: results from 7 prospective cohort studies', *Am J Clin Nutr*, vol. 113, no. 3, pp. 639–646, Mar. 2021, doi: 10.1093/ajcn/nqaa339.
[S15] S. Seino *et al.*, 'Dose-Response Relationships Between Body Composition Indices and All-Cause Mortality in Older Japanese Adults', *J Am Med Dir Assoc*, vol. 21, no. 6, pp. 726-733.e4, Jun. 2020, doi: 10.1016/j.jamda.2019.11.018.
[S16] T. I. A. Sørensen, P. Frederiksen, and B. L. Heitmann, 'Levels and changes in body mass index decomposed into fat and fat-free mass index: relation to long-term all-cause mortality in the general population', *Int J Obes (Lond)*, vol. 44, no. 10, pp. 2092–2100, Oct. 2020, doi: 10.1038/s41366-020-0613-8.
[S17] P. Srikanthan and A. S. Karlamangla, 'Muscle mass index as a predictor of longevity in older adults', *Am J Med*, vol. 127, no. 6, pp. 547–553, Jun. 2014, doi: 10.1016/j.amjmed.2014.02.007.
[S18] A. C. Tsai and T.-L. Chang, 'The effectiveness of BMI, calf circumference and mid-arm circumference in predicting subsequent mortality risk in elderly Taiwanese', *Br J Nutr*, vol. 105, no. 2, pp. 275–281, Jan. 2011, doi: 10.1017/S0007114510003429.
[S19] D. Kim, J. Lee, R. Park, C.-M. Oh, and S. Moon, 'Association of low muscle mass and obesity with increased all-cause and cardiovascular disease mortality in US adults', *J Cachexia Sarcopenia Muscle*, vol. 15, no. 1, pp. 240–254, Feb. 2024, doi: 10.1002/jcsm.13397.
[S20] C. Camargo Pereira, V. Pagotto, C. de Oliveira, and E. A. Silveira, 'Low muscle mass and mortality risk later in life: A 10-year follow-up study', *PLoS One*, vol. 17, no. 7, p. e0271579, 2022, doi: 10.1371/journal.pone.0271579.
[S21] F. Landi *et al.*, 'Estimated appendicular skeletal muscle mass using calf circumference and mortality: Results from the aging and longevity study in the Sirente geographic area (ilSIRENTE study)', *Exp Gerontol*, vol. 169, p. 111958, Nov. 2022, doi: 10.1016/j.exger.2022.111958.
[S22] C.-I. Li, C.-S. Liu, C.-H. Lin, S.-Y. Yang, T.-C. Li, and C.-C. Lin, 'Independent and joint associations of skeletal muscle mass and physical performance with all-cause mortality among older adults: a 12-year prospective cohort study', *BMC Geriatr*, vol. 22, no. 1, p. 597, Jul. 2022, doi: 10.1186/s12877-022-03292-0.
[S23] J. Liu, X. Jin, Z. Feng, and J. Huang, 'The association of central and extremity circumference with all-cause mortality and cardiovascular mortality: a cohort study', *Front Cardiovasc Med*, vol. 10, p. 1251619, 2023, doi: 10.3389/fcvm.2023.1251619.
[S24] M. Wu *et al.*, 'Associations of muscle mass, strength, and quality with all-cause mortality in China: a population-based cohort study', *Chin Med J (Engl)*, vol. 135, no. 11, pp. 1358–1368, Jun. 2022, doi: 10.1097/CM9.0000000000002193.
[S25] C.-S. Chang *et al.*, 'Optimal body composition indices cutoff values based on all-cause mortality in the elderly', *Exp Gerontol*, vol. 171, p. 112026, Jan. 2023, doi: 10.1016/j.exger.2022.112026.
[S26] Z. Ying *et al.*, 'Association of fat mass and fat-free mass with all-cause and cause-specific mortality in Asian individuals: A prospective cohort study', *Obesity (Silver Spring)*, vol. 31, no. 12, pp. 3043–3055, Dec. 2023, doi: 10.1002/oby.23878.
[S27] S. Moon *et al.*, 'Association of Appendicular Skeletal Muscle Mass Index and Insulin Resistance With Mortality in Multi-Nationwide Cohorts', *Journal of Cachexia, Sarcopenia and Muscle*, vol. 16, no. 2, e13811, 2025, doi:

10.1002/jcsm.13811.
[S28] Y. Cheng *et al.*, ‘Low appendicular skeletal muscle mass is associated with the risk of mortality among adults in the United States’, *Sci Rep*, vol. 15, no. 1, p. 9908, Mar. 2025, doi: 10.1038/s41598-025-94357-8.
[S29] S. Balogun *et al.*, ‘Prospective Associations of Low Muscle Mass and Function with 10-Year Falls Risk, Incident Fracture and Mortality in Community-Dwelling Older Adults’, *J Nutr Health Aging*, vol. 21, no. 7, pp. 843–848, 2017, doi: 10.1007/s12603-016-0843-6.
[S30] J. A. Batsis, S. Singh, and F. Lopez-Jimenez, ‘Anthropometric measurements and survival in older Americans: Results from the third National Health and Nutrition Examination Survey’, *J Nutr Health Aging*, vol. 18, no. 2, pp. 123–130, 2014, doi: 10.1007/s12603-013-0366-3.
[S31] J. W. Bea *et al.*, ‘Risk of Mortality According to Body Mass Index and Body Composition Among Postmenopausal Women’, *Am J Epidemiol*, vol. 182, no. 7,
pp. 585–596, Oct. 2015, doi: 10.1093/aje/kwv103.
[S32] P. M. Cawthon *et al.*, ‘Muscle Mass Assessed by the D3-Creatine Dilution Method and Incident Self-reported Disability and Mortality in a Prospective Observational Study of Community-Dwelling Older Men’, *J Gerontol A Biol Sci Med Sci*, vol. 76, no. 1, pp. 123–130, Jan. 2021, doi: 10.1093/gerona/glaa111.
[S33] Y. Chen *et al.*, ‘A prospective study of arm circumference and risk of death in Bangladesh’, *Int J Epidemiol*, vol. 43, no. 4, pp. 1187–1196, Aug. 2014, doi: 10.1093/ije/dyu082.
[S34] C.-L. Cheung, K. S. L. Lam, and B. M. Y. Cheung, ‘Evaluation of Cutpoints for Low Lean Mass and Slow Gait Speed in Predicting Death in the National Health and Nutrition Examination Survey 1999-2004’, *J Gerontol A Biol Sci Med Sci*, vol. 71, no. 1, pp. 90–95, Jan. 2016, doi: 10.1093/gerona/glv112.
[S35] L. Costanzo *et al.*, ‘Impact of Low Muscle Mass and Low Muscle Strength According to EWGSOP2 and EWGSOP1 in Community-Dwelling Older People’, *J Gerontol A Biol Sci Med Sci*, vol. 75, no. 7, pp. 1324–1330, Jun. 2020, doi: 10.1093/gerona/glaa063.
[S36] C. M. Dolan, H. Kraemer, W. Browner, K. Ensrud, and J. L. Kelsey, ‘Associations Between Body Composition, Anthropometry, and Mortality in Women Aged 65 Years and Older’, *Am J Public Health*, vol. 97, no. 5, pp. 913–918, May 2007, doi: 10.2105/AJPH.2005.084178.
[S37] B. Larsen *et al.*, ‘Muscle area and density and risk of all-cause mortality: The Multi-Ethnic Study of Atherosclerosis’, *Metabolism*, vol. 111, p. 154321, Oct. 2020, doi: 10.1016/j.metabol.2020.154321.
[S38] D. H. Lee *et al.*, ‘Predicted lean body mass, fat mass, and all cause and cause specific mortality in men: prospective US cohort study’, *BMJ*, vol. 362, p. k2575, Jul. 2018, doi: 10.1136/bmj.k2575.
[S39] R. Li *et al.*, ‘Associations of Muscle Mass and Strength with All-Cause Mortality among US Older Adults’, *Med Sci Sports Exerc*, vol. 50, no. 3, pp. 458–467, Mar. 2018, doi: 10.1249/MSS.0000000000001448.
[S40] P. D. Loprinzi and E. Frith, ‘Effects of Sedentary Behavior, Physical Activity, Frequency of Protein Consumption, Lower Extremity Strength and Lean Mass on All-Cause Mortality’, *J Lifestyle Med*, vol. 8, no. 1, pp. 8–15, Jan. 2018, doi: 10.15280/jlm.2018.8.1.8.
[S41] H. Oh *et al.*, ‘Adiposity and mortality in Korean adults: a population-based prospective cohort study’, *Am J Clin Nutr*, vol. 113, no. 1, pp. 142–153, Jan. 2021, doi: 10.1093/ajcn/nqaa258.

[S42] H. Wang, S. Hai, Y. Liu, Y. Liu, and B. Dong, 'Skeletal Muscle Mass as a Mortality Predictor among Nonagenarians and Centenarians: A Prospective Cohort Study', *Sci Rep*, vol. 9, no. 1, p. 2420, Feb. 2019, doi: 10.1038/s41598-019-38893-0.

[S43] S. G. Wannamethee, A. G. Shaper, L. Lennon, and P. H. Whincup, 'Decreased muscle mass and increased central adiposity are independently related to mortality in older men', *Am J Clin Nutr*, vol. 86, no. 5, pp. 1339–1346, Nov. 2007, doi: 10.1093/ajcn/86.5.1339.

[S44] L.-W. Wu *et al.*, 'Mid-Arm Circumference and All-Cause, Cardiovascular, and Cancer Mortality among Obese and Non-Obese US Adults: the National Health and Nutrition Examination Survey III', *Sci Rep*, vol. 7, p. 2302, May 2017, doi: 10.1038/s41598-017-02663-7.

[S45] G. Zong, Z. Zhang, Q. Yang, H. Wu, F. B. Hu, and Q. Sun, 'Total and regional adiposity measured by dual-energy X-ray absorptiometry and mortality in NHANES 1999-2006', *Obesity (Silver Spring)*, vol. 24, no. 11, pp. 2414–2421, Nov. 2016, doi: 10.1002/oby.21659.

[S46] A. Bernabe-Ortiz, R. M. Carrillo-Larco, R. H. Gilman, L. Smeeth, W. Checkley, and J. J. Miranda, 'Skeletal muscle mass and all-cause mortality: Findings from the CRONICAS cohort study', *Trop Med Int Health*, vol. 28, no. 2, pp. 107–115, Feb. 2023, doi: 10.1111/tmi.13844.

[S47] Y. Tabara, K. Setoh, T. Kawaguchi, and F. Matsuda, 'Skeletal muscle mass index is independently associated with all-cause mortality in men: The Nagahama study', *Geriatr Gerontol Int*, vol. 22, no. 11, pp. 956–960, Nov. 2022, doi: 10.1111/ggi.14491.

[S48] R. Knowles, J. Carter, S. A. Jebb, D. Bennett, S. Lewington, and C. Piernas, 'Associations of Skeletal Muscle Mass and Fat Mass With Incident Cardiovascular Disease and All-Cause Mortality: A Prospective Cohort Study of UK Biobank Participants', *J Am Heart Assoc*, vol. 10, no. 9, p. e019337, May 2021, doi: 10.1161/JAHA.120.019337.

[S49] L.-W. Wu *et al.*, 'Mid-arm muscle circumference as a significant predictor of all-cause mortality in male individuals', *PLoS One*, vol. 12, no. 2, p. e0171707, 2017, doi: 10.1371/journal.pone.0171707.
